# City-Scale, Source-Resolved Methane Inventories Reveal Drivers and Mitigation Pathways Across China’s Cities

**DOI:** 10.1021/acs.est.5c18654

**Published:** 2026-08-03

**Authors:** Li Zhang, Yifan Chen, Ke Wang, Jing Guo, Sen Liang, Pengcheng Wu, Haoyu Zhang, Jingyi Wu, Ying Cui, Chen Lyu, Hui Xu, Qunwei Wang, Bofeng Cai, Jinnan Wang, Linyan Li

**Affiliations:** † School of the Environment and Safety Engineering, 12676 Jiangsu University, Zhenjiang 212013, China; ‡ Jiangxi Provincial Key Laboratory of Greenhouse Gas Accounting and Carbon Reduction, Institute of Energy Research, Jiangxi Academy of Sciences, Nanchang 330096, China; § Department of Data Science, 53025 City University of Hong Kong, Hong Kong SAR 999077, China; ∥ School of Land Science and Technology, 113084 China University of Geosciences, Beijing 100083, China; ⊥ Center for Carbon Neutrality, 90404 Chinese Academy of Environmental Planning, Beijing 100043, China; # 74750 Chinese Academy of Natural Resources Economics, Sanhe City 101149, China; ¶ School of Public Health, 26469 Sun Yat-sen University, Guangzhou, Guangdong 510080, China; ∇ State Grid Shaanxi Electric Power Company Limited Research Institute, Xi’an 710065, China; ○ School of Environmental Science and Engineering, 12474 Shanghai Jiao Tong University, Shanghai Engineering Research Center of Solid Waste Treatment and Resource, Shanghai 200240, China; ⧫ School of Environmental and Chemical Engineering, 12596 Shanghai University of Electric Power, Shanghai 200090, China; †† College of Economics and Management, Nanjing University of Aeronautics and Astronautics, Nanjing 211106, China; ‡‡ Department of Infectious Diseases and Public Health, City University of Hong Kong, Hong Kong SAR 999077, China

**Keywords:** methane emissions, city-level inventory, source-resolved emissions, LMDI decomposition, multisector mitigation pathways

## Abstract

City-scale, source-resolved methane (CH_4_) inventories are needed in China because prefecture-level cities differ in energy systems, agricultural activities, and waste management. These differences lead to divergent dominant sources and mitigation needs. Yet city-level CH_4_ inventories across the energy, agriculture, and waste sectors remain scarce. This scarcity limits policy design, targeting, and evaluation. Here we develop a harmonized, annually consistent, multisector, source-resolved CH_4_ inventory for 339 prefecture-level cities in China during 2018–2024. We further use Logarithmic Mean Divisia Index (LMDI) driver attribution and scenario analysis to examine recent emission drivers and explore possible mitigation pathways. Over this period, national anthropogenic CH_4_ emissions increased by 2.61%, with regional heterogeneity shaped by differences in energy dependence, livestock management, and waste treatment. LMDI decomposition identifies economic activity as the dominant positive driver. Emission-intensity effects alternated between mitigating and reinforcing impacts, while structural effects were minor and population effects varied across space and time. Scenario simulations suggest that integrated multisector strategies could reduce national CH_4_ from 61.69 Mt in 2024–51.26 Mt in 2030 and 22.26 Mt in 2060, corresponding to a 63.9% reduction. These reductions are primarily driven by energy-sector measures, complemented by improvements in agriculture and waste management. This data set and attribution inform city-specific mitigation planning and source targeting, support monitoring, reporting, and verification, and provide a baseline for benchmarking progress toward national methane targets.

## Graphical abstract

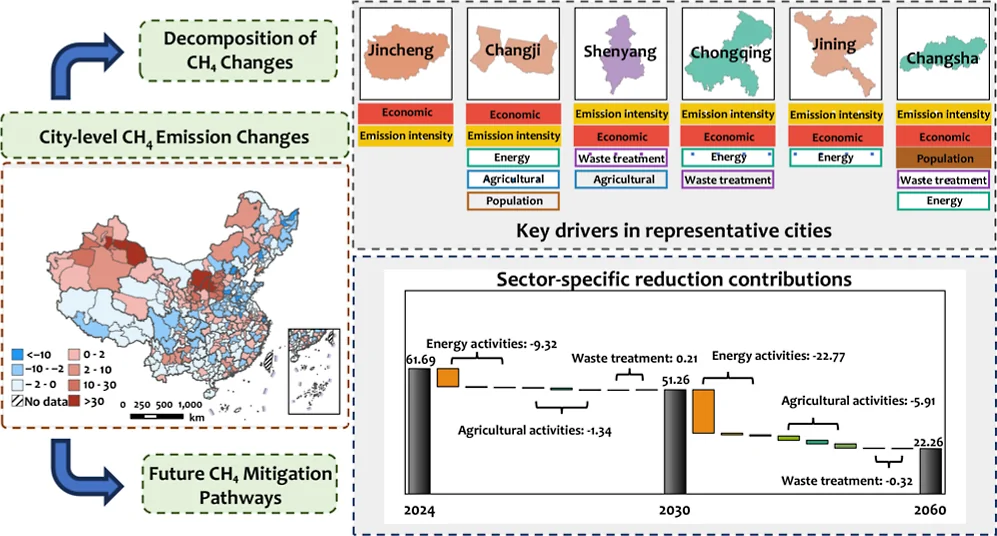

## Introduction

Methane (CH_4_) is the second most important anthropogenic greenhouse gas after carbon dioxide (CO_2_), with a 20 year global warming potential ∼80–83 times that of CO_2_.^1^ Anthropogenic activities account for about 60% of global methane emissions, mainly from agriculture (∼40%), fossil fuels (∼35%), and waste (∼20%).^2^ Due to its high warming potency and relatively short atmospheric lifetime of ∼12 years, methane mitigation is widely regarded as one of the most effective strategies for slowing near-term climate warming.^3,4^ Reflecting this urgency, the Global methane pledge launched at COP26 commits to reducing anthropogenic methane emissions by at least 30% from 2020 levels by 2030, with the potential to avoid up to 0.2 °C of warming by midcentury.^5^

Global assessments typically classify anthropogenic methane emissions into three major sectors: energy production, agriculture, and waste management.^1,2,6^ Methane emissions are quantified using two complementary approaches. Bottom-up inventories estimate emissions from activity data and emission factors,^7–11^ while atmospheric observations provide independent constraints based on measurements.^12,13^ Satellite instruments such as TROPOMI have further improved the detection of methane enhancements over cities and source regions.^14,15^ However, such observations are difficult to translate into city- and sector-resolved estimates without harmonized activity data, consistent sector definitions, and reliable allocation to administrative units. Bottom-up inventories also remain affected by incomplete or outdated activity data, uncertain emission factors, differences in system boundaries, and spatial allocation methods.^16,17^ These issues can produce divergent subnational estimates and make source attribution, annual trend detection, and policy evaluation more difficult.^13,17,18^ This challenge is particularly relevant in an urban setting, where emissions are shaped by heterogeneous energy systems, agricultural activities, and waste infrastructure, and where mitigation measures are often implemented and evaluated. A temporally consistent, multisector city-level inventory is therefore needed to compare emissions across cities and identify dominant sources. Such an inventory can also support monitoring, reporting, and verification (MRV) applications.^8,19^

Within this context, China is a major source of anthropogenic methane, with emissions mainly associated with energy production, agriculture, and waste management.^2^ Methane estimates for China have been developed through global gridded inventories, national and provincial inventories, sector-specific studies, and city-level studies, each with different uses and limitations. Global and gridded inventories provide broad spatial coverage, but their sector definitions and spatial allocation methods are not primarily designed for prefecture-level methane accounting in China.^6,16,17^ National and provincial studies are useful for estimating total emissions and broad regional patterns, but they can mask large differences among cities within the same province.^7^ Sector-specific studies have improved understanding of methane emissions from coal mining, rice cultivation, livestock, landfills, and wastewater, but they do not provide a consistent multisector basis for comparing cities.^9–11,20–25^ Existing city-level studies provide useful local evidence, but many are based on one year, selected cities, or selected sources.^8,26–28^ China’s recent emphasis on methane and non-CO_2_ greenhouse gas mitigation further increases the need for comparable city-level evidence to support source control and progress tracking.^29,30^

To address this gap, we developed a harmonized annual methane inventory for 339 Chinese prefecture-level cities during 2018–2024, with source resolution across the energy, agriculture, and waste sectors. The inventory provides a consistent basis for comparing cities, tracking annual changes, and identifying dominant emission sources. We then use Logarithmic Mean Divisia Index (LMDI) decomposition to examine recent emission drivers and apply scenario analysis to explore possible mitigation pathways. By linking city-level accounting with driver attribution and pathway analysis, this study provides a data basis for identifying priority sources, tracking emission changes, and planning methane mitigation across Chinese cities.

## Results

### Spatiotemporal Patterns of China’s City-Level Methane Emissions

Our national totals were consistent with China’s official national greenhouse gas inventory for overlapping years, and only minor differences were observed (Table S1). Estimated uncertainties across sectors were broadly in line with previous studies,^26^ ranging from 12% to 22% across the assessed subsectors, with wastewater treatment showing the largest overall uncertainty (Table S2). Nationally, China’s total methane (CH_4_) emissions remained broadly stable at around 60 Mt yr^–1^ during 2018 to 2024, with modest interannual variability, including a local minimum in 2021 and a slight increase toward 2024 (Figure 1a). Although the national total changed little, sectoral trends diverged, with emissions from energy activities increasing overall and those from agriculture and waste management declining, resulting in a net increase of 1.56 Mt (2.61%) from 2018 (60.13 Mt) to 2024 (61.69 Mt), driven primarily by the energy sector.

**Figure 1:**
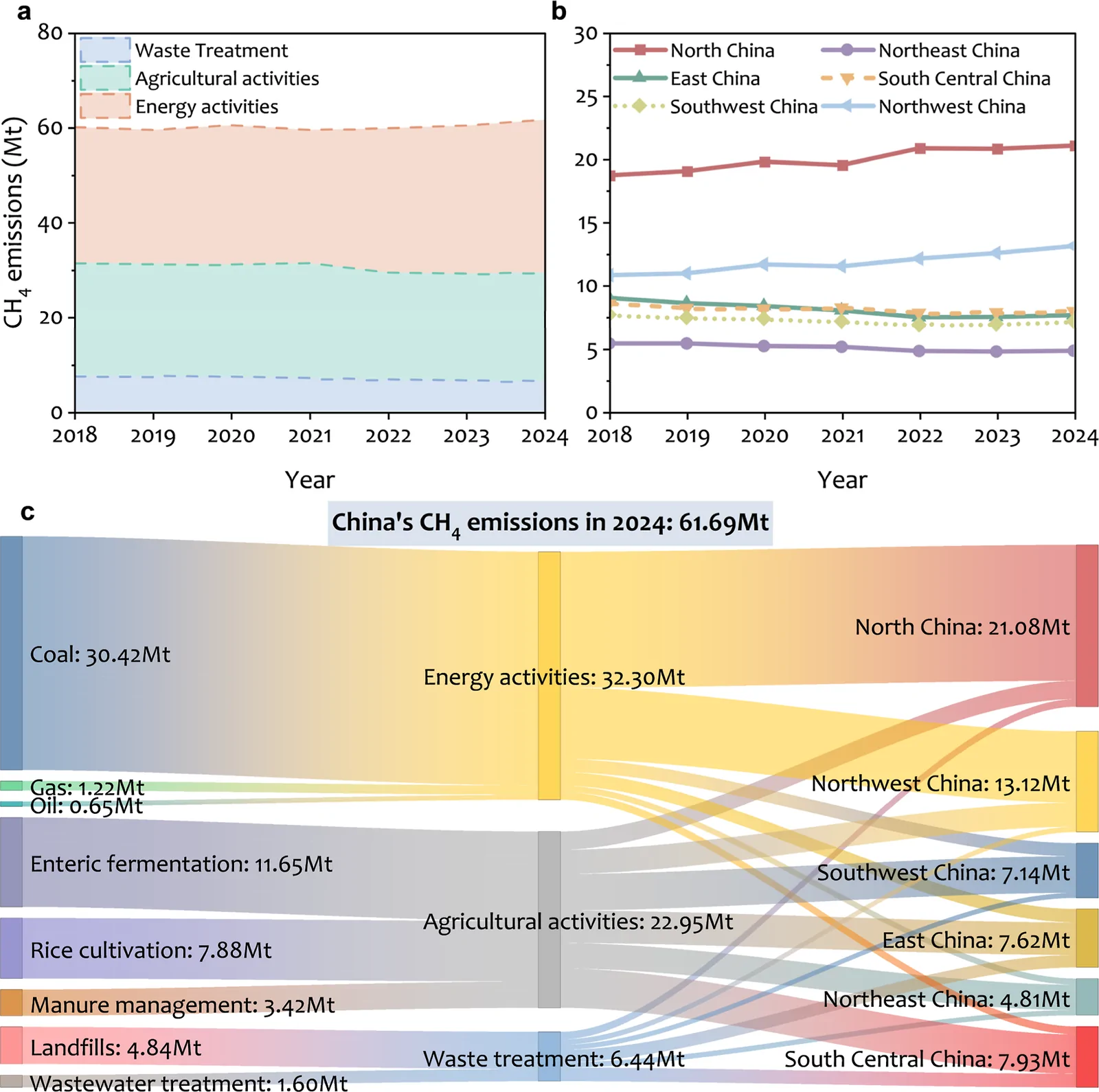
Spatiotemporal patterns of CH_4_ emissions in China (2018–2024). (a) Trends of national CH_4_ emissions from 2018 to 2024 by sector. (b) CH_4_ emission trends from 2018 to 2024 across six regions of China, showing regional differences over time. (c) Sectoral contributions to national CH_4_ emissions in 2024.

Regionally, emissions increased in North China and Northwest China but declined in East China, South Central China, Southwest China, and Northeast China (Figure 1b). Sectoral decomposition shows that the increases in North China and Northwest China were dominated by energy activities, whereas declines elsewhere reflected reductions across multiple sectors, with region-specific dominant contributors. Waste treatment accounted for the largest share of the decline in East China, agricultural activities together with waste treatment dominated the declines in South Central and Southwest China, and energy activities dominated the decline in Northeast China (Figure S1 and Table S3). Overall, these results indicate the growing importance of energy-related methane emissions and persistent regional heterogeneity across China. A cross-inventory comparison at the national and provincial levels, including sectoral benchmarking, is provided in the Supporting Information (Tables S4–S6).

In 2024, China’s total methane emissions reached 61.69 Mt, with energy (52.36%, 32.30 Mt) and agriculture (37.20%, 22.95 Mt) as the dominant contributors (Figure 1c). Coal mining alone accounted for 30.42 Mt and was the largest single source. Agricultural emissions were driven mainly by intestinal fermentation (11.65 Mt) and rice cultivation (7.88 Mt), reflecting the scale of livestock and paddy farming.

City-level emission profiles reveal clear source-specific patterns (Figure 2a). Energy-related methane emissions were concentrated in Xinjiang, Shanxi, Shaanxi, and the junction of Hebei, Henan, Shandong, and Anhui provinces. In 2024, 65 cities derived more than half of their methane emissions from energy activities. Extreme reliance (≥95% of total emissions) was observed in several coal-producing prefectures, including Jincheng and Shuozhou in Shanxi and Ordos in Inner Mongolia (see Tabel S7 for the full list). By contrast, agricultural activities accounted for more than 50% of emissions in 220 cities, reflecting the sustained prominence of livestock husbandry and rice cultivation.

**Figure 2:**
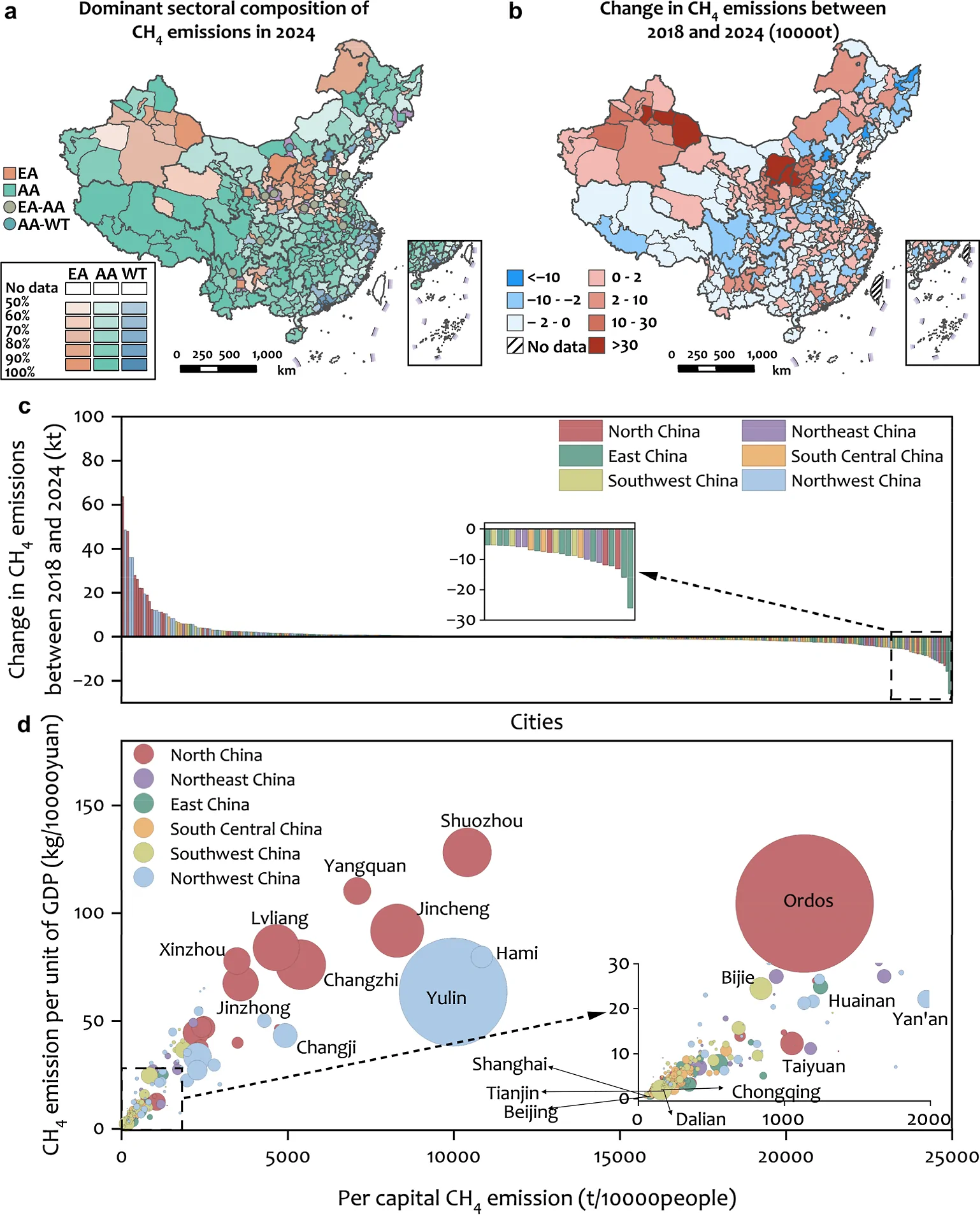
Spatial and structural patterns of CH_4_ emissions across Chinese prefecture-level cities. (a) Sectoral composition of CH_4_ emissions in 2024, highlighting the relative contributions of energy activities, agricultural activities, and waste treatment. (b) Changes in city-level CH_4_ emissions between 2018 and 2024. (c) Net change in city-level CH_4_ emissions from 2018 to 2024, with prefecture-level cities on the horizontal axis ordered from the largest increase to the largest decrease and color-coded by six regions. (d) Relationship between per capita GDP, emission intensity (per unit GDP), and total CH_4_ emissions (bubble size).

Furthermore, waste-related emissions accounted for the majority of total methane emissions in 38 cities, primarily in economically developed, densely populated coastal agglomerations. These include large parts of the Pearl River Delta and Yangtze River Delta, with representative examples including Guangzhou, Shenzhen, Shanghai, and Suzhou. Several inland hubs such as Beijing and Chengdu also exhibited waste-dominated profiles (see Table S8 for the complete list of waste-dominated cities). This contrast between resource-intensive energy hubs and agrarian or waste-driven systems underscores the need for regionally differentiated mitigation strategies.

At a city level, methane emissions exhibited pronounced spatial heterogeneity (Figure 2b). Most prefecture-level cities experienced relatively small net changes (−100 to 100 kt), but a limited number showed substantial shifts. Large reductions (>100 kt) were confined to a small group of major cities with substantial industrial activity, including representative examples such as Jining, and Huainan, as well as megacities Shanghai and Beijing. By contrast, large increases (>100 kt) were concentrated in coal- and gas-producing prefectures, with representative examples including Ordos, Lvliang, and Yulin. The full lists of cities with the largest increases and decreases are provided in Table S9. Regionally, these city-level changes resulted in the largest aggregate increases in North and Northwest China, alongside modest net declines in East China (Figure 2c). For example, in Shandong Province, Jining registered one of the steepest emission reductions, consistent with closures of coal-coking plants and broader energy-sector reforms during the study period.

Comparative analyses of total emissions, per capita emissions, and emissions intensity per unit of GDP further highlight these disparities (Figure 2d). Coal-dependent prefectures, including Ordos, Shuozhou, and Yulin, ranked high across all three metrics, reflecting large fugitive emissions, relatively low population density, and weaker economic output. A second category includes prefectures with modest total emissions but disproportionately high per capita emissions and per-unit GDP emissions intensity, consistent with livestock-dominated emission structures; examples include Hami and Changji Hui Autonomous Prefecture. In contrast, megacities, including Beijing, Shanghai, and Guangzhou, exhibit high total emissions but comparatively low per capita and per-unit-GDP CH_4_ emissions, due to their large populations. Finally, some coastal and medium-sized cities, such as Xiamen, Weihai, and Zhoushan, exhibit consistently low emissions across all three measures.

### Regional Drivers of City-Level Methane Emissions

Between 2018 and 2024, interannual variations in national methane emissions reflected the combined influence of population, economic activity, emission intensity, and sectoral structural changes (Figure S2). Across all subperiods, the economic effect emerged as the dominant positive driver of emission growth. In the LMDI decomposition, this effect reflects GDP per capita growth. Improvements in emission intensity, measured as changes in CH_4_ emissions per unit GDP, consistently exerted substantial downward pressure. This downward effect was particularly pronounced from 2020 to 2021, when emission-intensity improvements more than offset the economic effect and yielded a net decline in emissions. The population effect, defined as changes in total population, remained comparatively small throughout. Sectoral structural components exhibited greater heterogeneity. Expansion of energy-related activities systematically increased emissions, whereas changes in agricultural activities and waste treatment exerted only minor contributions. Taken together, these countervailing forces resulted in relatively modest net changes in national methane emissions from 2018 to 2024, despite pronounced shifts in the underlying drivers.

Regional patterns revealed pronounced spatial heterogeneity (Figure 3). For readability, Figure 3a–f display a screened subset of cities using region-specific cutoffs, and full thresholds and city lists are provided in Table S10. In North China (Figure 3a), Ordos (Inner Mongolia) recorded the largest emission increase within the region, driven simultaneously by positive population, economic, and emission-intensity effects. Most cities in Shanxi exhibited increases in methane emissions predominantly driven by the economic effect, whereas reductions in emission intensity and, to a lesser extent, population exerted countervailing downward pressures that were insufficient to offset this upward trajectory. In Northwest China (Figure 3b), emissions in most cities remained stable. Yulin emerged as a hotspot of growth, where positive economic effects outweighed the negative contribution from emission intensity. Other cities with rising emissions, such as Hami and Changji (Xinjiang), were characterized by positive contributions from the population, economic, and emission-intensity effects, alongside slightly positive energy activities and minor negative agricultural and waste effects.

**Figure 3:**
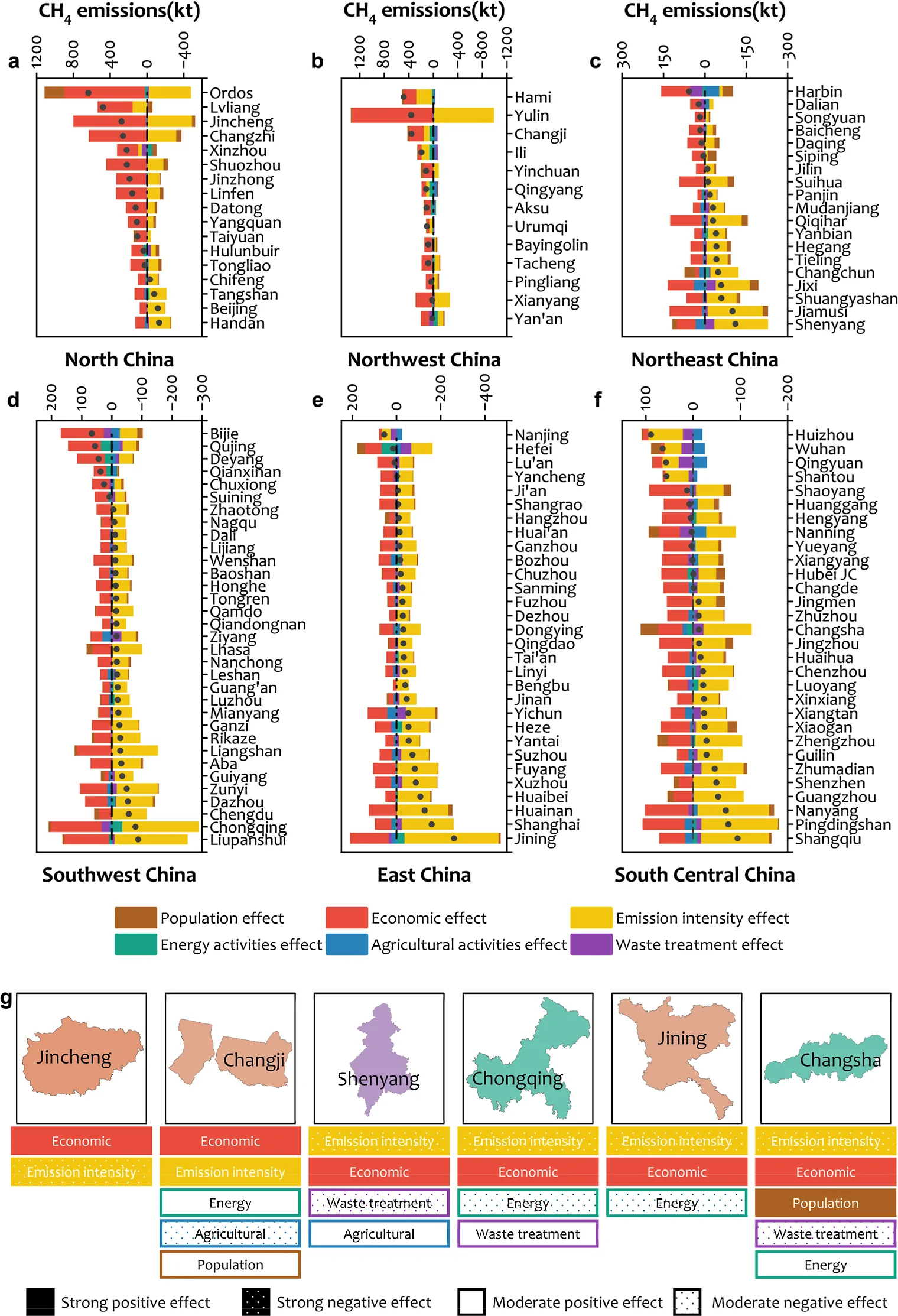
LMDI decomposition of CH_4_ emissions across China’s six regions, with illustrative city examples (2018–2024). Panels a–f show LMDI decomposition of CH_4_ emissions for six regions. For readability, panels a–f display a screened subset of cities in each region. Cities were included if either the absolute total effect or any of the six driver contributions exceeded the region-specific cutoff in absolute value, and included cities are ranked by the magnitude of the net change in CH_4_ emissions during 2018–2024. Full screening thresholds and city lists are provided in Table S10. Bars show the contributions of the population, economic activity, emission intensity, and three sectoral structural components, including energy activities, agricultural activities, and waste treatment. Panel g presents six representative cities, one from each region, with their LMDI driver decompositions. These cities were selected to illustrate distinct dominant driver profiles observed across regions rather than being chosen solely by emission level. For each city, driver effects are classified as strong (>15%) or moderate (5–15%), and effects are ordered vertically by decreasing absolute magnitude.

In the Northeast (Figure 3c), most cities like Jiamusi, Jixi, and Qiqihar experienced net reductions, with negative contributions from the emission-intensity and population effects outweighing positive contributions from the economic effect. By contrast, in Harbin, methane emissions increased overall, driven mainly by the economic effect and wastewater-related activities. Emission-intensity and energy-activity effects were relatively weak, while agricultural activity and population exerted negative contributions that only partially offset this upward pressure. In Southwest China (Figure 3d), Liupanshui achieved substantial reductions through the emission-intensity effect. Chongqing, Bijie, Qujing, Deyang, and Qianxinan all exhibited marked multifactor contributions, reflecting a complex interplay among economic activity, emission intensity, and sectoral structural effects in shaping city-level methane emissions. Here, sectoral structural effects refer to changes in the relative contributions of energy, agriculture, and waste activities. In Chongqing, this configuration was associated with a net reduction in emissions, whereas in Bijie, Qujing, Deyang, and Qianxinan it coincided with net increases in emissions.

In East China (Figure 3e), cities in Shandong, such as Jining, achieved notable reductions driven by the emission-intensity effect linked to industrial restructuring, while Shanghai further reduced emissions through the emission-intensity effect associated with advances in waste management. In South Central China (Figure 3f), most prefecture-level cities clustered around zero, indicating relatively modest net changes in methane emissions. In many cities, strong positive economic effects were largely counterbalanced by substantial negative emission-intensity effects, yielding near-neutral outcomes. By contrast, Shenzhen, Guangzhou, Nanyang, Pingdingshan and Shangqiu exhibited more pronounced emission reductions, where improvements in emission intensity dominated over economic expansion and other sectoral drivers. Population and other activity effects were generally small across the region.

Collectively, these patterns underscore the substantial spatial heterogeneity of city-level methane emissions across China during 2018–2024, reflecting the varying contributions of the population, economic, and emission-intensity effects, and structural effects.

### City-Level Methane Mitigation under Integrated Scenario Pathways

To translate the empirical patterns into actionable policy, we evaluate city-level methane mitigation under integrated scenario pathways. National methane emissions are projected to decline from 61.69 Mt in 2024 to 51.26 Mt in 2030, and further to 22.26 Mt by 2060, representing an overall reduction of nearly 63.9% via integrated multisector strategies (Figure 4a). The trajectory exhibits two distinct phases: rapid near-term abatement between 2024 and 2030 (−10.43 Mt), followed by sustained long-term reductions through 2060 (−29 Mt).

**Figure 4:**
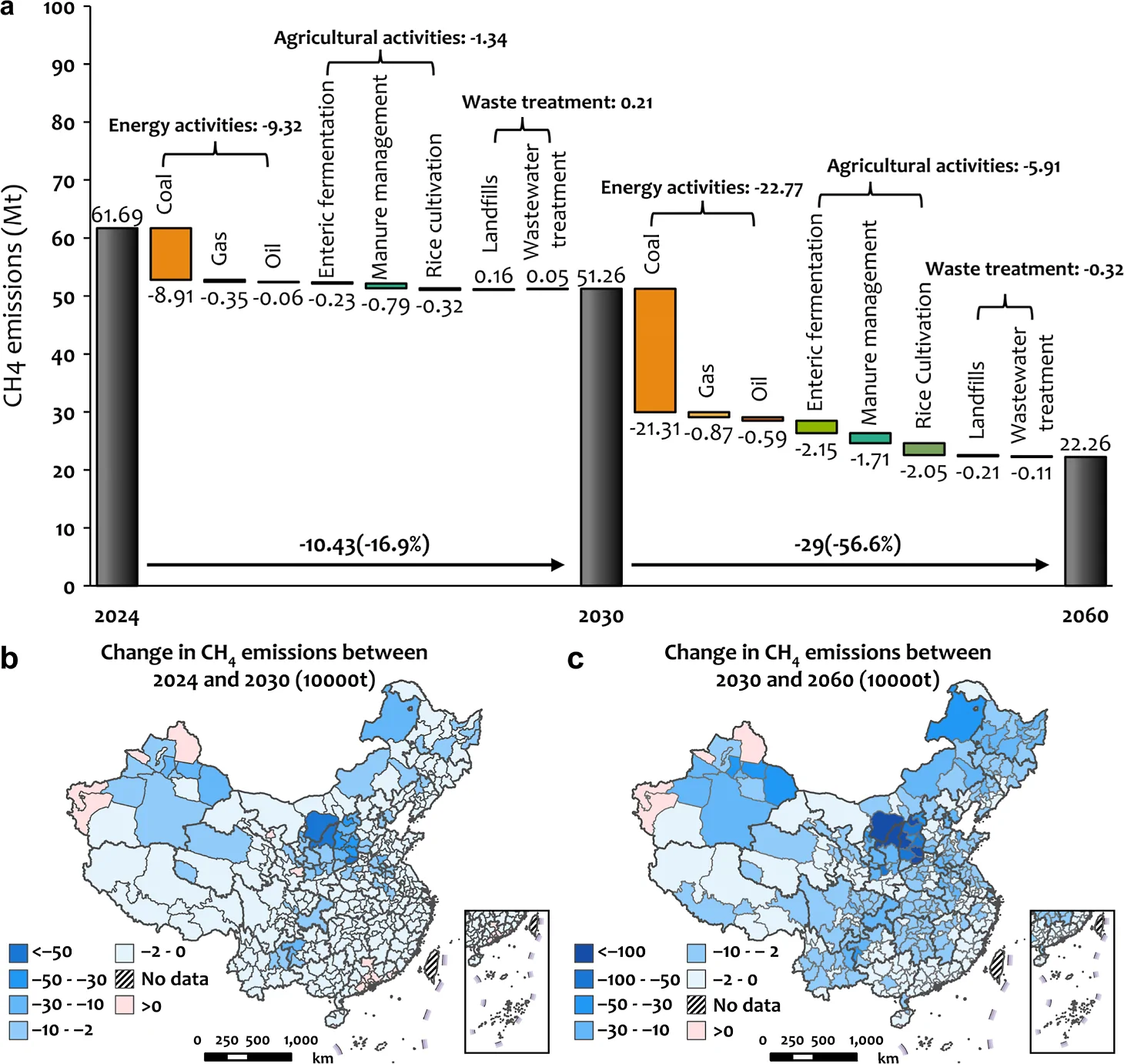
Sectoral and spatial patterns of projected CH_4_ emission reductions in China under combined mitigation scenarios. (a) National CH_4_ emissions in 2024, 2030, and 2060 and their sectoral decomposition. Colored bars show the contributions of energy activities (coal, gas, oil), agricultural activities (enteric fermentation, manure management, rice cultivation) and waste treatment (landfills, wastewater treatment) to changes in emissions between 2024–2030 and 2030–2060. Numbers above the sector groups indicate total sectoral contributions (Mt), and arrows denote net national changes over each period (Mt and % relative to 2024 for 2024–2030 and to 2030 for 2030–2060). (b,c) Spatial distribution of projected changes in city-level CH_4_ emissions between 2024 and 2030 (b) and between 2030 and 2060 (c). Blue shades indicate emission reductions, pink shades indicate increases and hatched areas denote cities with no data.

At the sectoral level, energy-related activities overwhelmingly dominate mitigation potential, primarily through coal mine methane recovery and reduced fossil fuel dependence. Agricultural and waste interventions contribute smaller amounts. Improvements in livestock and manure management, and in rice cultivation, drive reductions in agricultural emissions. Waste reductions are achieved via enhanced solid waste disposal and sewage treatment (Figure 4a). The combined share of agriculture and waste increases from 10.8% during 2024 to 2030 to 21.5% during 2030 to 2060, but their absolute contribution remains much smaller than that of energy.

Spatially, mitigation potential is concentrated in energy-intensive northern and northwestern cities, including Ordos (Inner Mongolia), Yulin (Shaanxi), and multiple coal-producing municipalities in Shanxi (Figure S3). Agricultural reductions are more dispersed, with notable contributions from Chongqing, Liangshan Prefecture (Sichuan), and several northeastern cities, such as Qiqihar, Jiamusi, and Harbin (Figure S4). Waste-related reductions are focused in major cities, including Beijing, Shanghai, Shenyang, Harbin, Changchun, Chongqing, and Hangzhou, reflecting city-level infrastructure capacity (Figure S5).

The ranking of top-reducing cities remains largely consistent across both phases, with energy-intensive regions maintaining absolute dominance (Figure 4b,c). These patterns highlight pronounced spatial and sectoral heterogeneity in mitigation potential: near-term and long-term reductions are primarily driven by energy interventions in key producing cities, whereas agriculture and waste measures contribute supplementary reductions across other cities. These findings underscore the need for city-level differentiated mitigation policies that prioritize energy-sector reforms in major producing cities while supporting agricultural and waste management improvements in other cities to achieve long-term methane reduction targets.

## Discussion

By developing an annually consistent methane inventory for 339 Chinese prefecture-level cities from 2018 to 2024, this study provides a comparable basis for examining methane emissions across cities, sectors, and years. The inventory also supports driver attribution through LMDI decomposition and the assessment of mitigation pathways under different scenarios. National anthropogenic methane emissions increased by 2.61% during 2018 to 2024, but this modest national change masked clear regional and sectoral differences associated with energy structure, livestock management, and waste management systems. Such sectoral and city-level differences indicate that national or provincial totals provide only a partial view of methane emission heterogeneity in China. A city-level, source-resolved inventory therefore provides a more specific basis for identifying dominant sources and assessing mitigation pathways.

Although the national total remained near 60 Mt yr^–1^ from 2018 to 2024, this apparent stability reflects offsetting sectoral changes rather than uniform stagnation. Increases in energy-related methane emissions were partially offset by declines in agriculture- and waste-related emissions. This pattern is consistent with the LMDI decomposition, which showed that the economic activity effect exerted persistent upward pressure, whereas the emission-intensity effect varied across years. The energy sector increase is also consistent with the continued importance of coal and the concentration of methane–intensive activities in key producing regions. In these regions, fugitive and ventilation emissions from coal mining can dominate local methane profiles.^31,32^ In contrast, the downward trajectories in agriculture and waste are consistent with sectoral mitigation guidance and inventory methods related to manure management, landfill gas capture, and wastewater treatment.^29,33^ These divergent sectoral trends explain why the national total changed only modestly over the study period.

Regional divergence further shows that methane emissions are closely linked to the geography of energy production. Large increases were concentrated in coal- and gas-producing prefectures, where emission trajectories are related to extraction intensity and the implementation of recovery, utilization, and leak control measures. Large declines were more common in major industrial and urban centers, where industrial restructuring and improvements in municipal waste and wastewater systems may have contributed to lower emissions.^29,31,32^ To assess consistency with existing estimates, we benchmarked our national and provincial aggregates against established bottom-up inventories (Tables S4–S6). Provincial patterns were broadly consistent across inventories, whereas differences in absolute totals mainly reflected sector coverage, emission-factor parametrization, and spatial allocation choices, especially for energy-related sources. These contrasts suggest that mitigation priorities may need to differ across prefectures, with fugitive emission control emphasized in key producing regions and continued infrastructure and management upgrades in urbanizing regions.

China’s carbon peaking and neutrality strategy now gives greater attention to non-CO_2_ greenhouse gases, including methane. Recent policy documents, such as the Methane Emissions Control Action Plan^29^ and China’s Updated Nationally Determined Contributions,^30^ call for improving methane MRV and stronger sector-specific mitigation. In this context, city-level methane accounting can help link national targets with local source control. The inventory developed here provides a basis for identifying major sources, comparing mitigation priorities across cities, and tracking changes under a consistent framework. If incorporated into a standardized MRV system, such inventories could improve data comparability and transparency, and support progress assessment under China’s 2035 non-CO_2_ reduction target.

Sector-specific measures remain important for future methane reductions. The energy sector is the largest source in the current inventory, and emission control in this sector depends mainly on coal mine methane recovery, leak detection, and gas utilization.^34^ Agricultural mitigation can involve improved manure management and optimized rice cultivation, while waste-sector reductions depend on landfill gas recovery, improved wastewater treatment, and integrated waste–management transitions.^20,35^ Linking these measures to a consistent MRV framework would allow cities to track sources, activities, and mitigation progress more clearly.

Globally, methane mitigation is receiving greater attention through initiatives such as the Global Methane Pledge and the enhanced transparency framework under the Paris Agreement.^5^ Many countries still face difficulties in maintaining consistent monitoring systems and translating national targets into subnational actions.^32^ In this context, the city-level, source-resolved accounting framework developed here may provide a useful reference for improving inventory detail, identifying dominant sources, and supporting MRV-related assessment. The heterogeneity observed across Chinese prefectures shows that mitigation opportunities and implementation conditions can differ substantially within one country. This supports the value of subnational evidence for prioritizing actions and tracking progress. Although this study does not assess international burden sharing or cooperation outcomes, its methods and data structure could support cross-country benchmarking when comparable subnational inventories become available. They may also inform technical exchange on inventory development, data standardization, and verification practices.^36^

Several limitations should be noted. First, although multiscale data calibration, cross-inventory benchmarking, and standardized accounting methods were used to improve consistency, uncertainty remains because long-term, full-sector historical data are limited at the city level. This limitation affects both city-scale model calibration and long-term scenario analysis. Second, the LMDI decomposition was conducted only for 2018–2024, so the identified drivers may partly reflect short-term shocks during this period, including the pandemic, the subsequent resumption of work and production, and temporary changes in energy-related activities. These results should therefore be interpreted as recent-period drivers rather than stable long-term mechanisms. Third, the scenario analysis depends on assumptions about activity levels, sectoral transitions, and mitigation technologies. The projected pathways should therefore be interpreted as possible mitigation pathways rather than deterministic forecasts. Future work should expand the time coverage of city-level methane inventories, reconstruct longer historical series using multiple data sources, refine city-specific parameters with local monitoring and statistical data, and update scenario settings as mitigation policies and technologies evolve.

## Methods

This study on city-scale methane emission accounting encompasses key sectors including energy, agriculture, and waste management. Specifically, the energy sector involves coal mining, oil and gas extraction. The waste management sector comprises landfill operations and wastewater treatment. The agricultural sector includes livestock and poultry farming, and rice cultivation. The specific accounting methodologies and data foundations for methane emissions across these sectors are described below. Activity data and emission factors were compiled from official statistics, sectoral reports, and published literature. Detailed source information for each emission category is summarized in Table S11.

### Calculation of the CH_4_ Emissions from the Energy Sector

#### Fugitive CH_4_ Emissions from Coal Production

Based on the basic data of 3137 coal mines across the country (including geographical coordinates, mine type, production capacity, mining depth and emission coefficient), we developed a methane emission accounting model applicable to coal mining operations. We combined point-source coal mining area production data and imposed activity level data constraints at the provincial and national levels. The emission quantity accounting method mainly follows the method recommended by the Intergovernmental Panel on Climate Change (IPCC) to quantify the fugitive methane emissions related to coal mining.

1 $$E_{FC,i,y}=EF_{FC,i,y}\times AP_{FC,i,y}$$

where *E*_FC,*i*,*y*_ stands for fugitive CH_4_ emissions from coal production (g); EF_FC,*i*, *y*_ represents emission factor regarding coal production (g CH_4_/g coal), which derived from the provincial greenhouse gas emission accounting guidelines; AP_FC,*i*,*y*_ is coal production of unit *i* in year *y*. The data on each enterprise’s activity level is sourced from the global energy monitor (GEM) database and the China’s high spatial resolution emission grid database (CHRED), and is constrained at the provincial and national levels. The data on activity levels at the provincial and national levels are sourced from statistical yearbooks and the National Bureau of Statistics.

#### Fugitive CH_4_ Emissions from Oil and Gas Production

This study takes the spatial location information and emission factor data of 32 oil and gas fields across the country as the core basis, matches the operation management data and annual public reports of oil and gas enterprises, and completed the provincial-scale detailed decomposition of national oil and gas production data. Based on this, a methane emission accounting model for the entire process of oil and gas development was constructed. For the fugitive emissions during the oil and gas production process, this study conducted itemized accounting for the two major systems of petroleum and natural gas according to the entire chain subprocesses: the petroleum system accounted for the boundaries covering the entire process from crude oil exploration, onshore and offshore crude oil production, multiple transportation methods (including pipelines, oil tankers, oil tank trucks, and railway transportation); the natural gas system accounted for the boundaries covering the entire process from natural gas exploration, onshore and offshore natural gas production, purification processing, trunk line transmission, storage, and terminal distribution.

2 $$E_{OG,i,p,y}=AP_{OG,i,p,y}\times EF_{OG,i,p,y}$$

where *E*_OG,*i*,*p*,*y*_ stands for fugitive CH_4_ emissions from oil and gas production (g) from process *p* in plant *i*; EF_OG,*i*,*p*,*y*_ refers to emission factor in terms of oil and gas production (g CH_4_/g oil or g CH_4_/m^3^ gas), which is derived from IPCC reports^16^ and the provincial greenhouse gas emission accounting guidelines; AP_OG,*i*,*p*,*y*_ is activity data for each stage of the crude oil and natural gas systems, which is derived from the annual reports and annual summaries of various oil and gas companies.

### Calculation of the CH_4_ Emissions from the Agriculture Sector

#### CH_4_ Emissions from the Livestock and Poultry Farming Sector

Using national livestock farm information from the enterprise registration database of the China Administration for Industry and Commerce (821,974 farms covering pigs, sheep, cattle, horses, camels, mules, donkeys, poultry and other livestock categories), we developed a methane emission accounting model for livestock production. The model explicitly distinguishes production systems—including large-scale intensive operations, smallholder/scattered farming and grazing-based systems—to capture their differing impacts on CH_4_ emissions from enteric fermentation and manure management.^37^ The monthly national livestock inventory, slaughter volume, and product wholesale prices were used as the constraints and data verification basis for the model.

#### Animal Intestinal Fermentation

CH_4_ emissions from enteric fermentation at the plant level are calculated by multiplying the population of various livestock species—such as sheep, swine, horses, cattle, camels, mules, and donkeys—by their respective emission factors.

3 $$E_{FE,i,a,y}=EF_{FE,i,a,y}\times AP_{FE,i,a,y}$$

where *E*_FE,*i*,*a*,*y*_ denotes the CH_4_ emissions from enteric fermentation (g) generated from animal type *a* in plant *i* in year *y*; EF_FE,*i*,*a*,*y*_ represents the emission factor (g/heads) of related CH_4_ emission sources, which is primarily derived from official source and related research;^8,17,27,38^ AP_FE,*i*,*a*,*y*_ refers to the population (in heads) of different livestock species. The number of livestock farms at the plant scale was determined using the plant’s registered capital as the proxy allocation indicator. This was achieved through a detailed weight decomposition calculation based on the refined statistical data of livestock farming at the municipal level. The city-level basic data for livestock used in the calculation originated from the “China Agricultural Yearbook” and “China Livestock Industry and Veterinary Medicine Yearbook” from 2018 to 2024, as well as the provincial and municipal statistical yearbooks during the same period, and the annual national economic and social development statistical bulletins of each municipality.

#### Manure Management

CH_4_ emissions from manure management at the plant-specific levels refer to the CH_4_ released during the storage and treatment processes of livestock manure before it is applied to agricultural soils. The sources of these emissions encompass various animal categories, including pigs, nondairy cattle, buffalo, dairy cattle, sheep, horses, camels, mules, donkeys, and poultry. For each type of livestock, CH_4_ emissions from manure management are calculated by multiplying the species-specific animal population by the corresponding manure management emission factors, following the method outlined below.

4 $$E_{MM,i,a,y}=EF_{MM,i,a,y}\times AP_{MM,i,a,y}$$

where *E*_MM,*i*,*a*,*y*_ refers to CH_4_ emissions from manure management (g); EF_MM,*i*,*a*,*y*_ represents the associated emission factor (g/heads), which is derived from the provincial greenhouse gas emission accounting guidelines; AP_MM,*i*,*a*,*y*_ represents the livestock (heads) of different animal types. The calculation method for the activity level of its plant–level activities is the same as that for intestinal fermentation.

### CH_4_ Emissions from Rice Cultivation

Monthly CH_4_ emissions from rice cultivation are estimated by integrating high-resolution spatial activity data with temporal vegetation indices to characterize crop growth cycles. The calculation distinguishes between single-cropping and double-cropping rice systems.

#### Spatial Activity Data

Annual baseline emissions are first derived based on the specific cultivation area (*A*_*i*,*k*_) of single- and double-cropping rice. These areas are extracted from an existing 30 m resolution land cover classification map.^39^ Since this data set provides preclassified mapping of rice types with validated accuracy, no further classification or accuracy assessment was conducted in this study. The annual emission factors (EF_*k*_) are set as 236.7 kgCH_4_/ha for single-cropping rice and 241.0 kgCH_4_/ha for double-cropping rice.

#### CH_4_ Emissions Estimation

These annual emissions are temporally allocated to each month using the normalized difference vegetation index (NDVI) strictly as a proxy for seasonal physiological activity and methanogenesis intensity, not for spatial classification. The monthly CH_4_ emissions for city *i*, crop type *k*, in month *m* are calculated as follows.

5 $$E_{rice,i,k}=\sum_{i,m}\left(A_{i,k}\times {EF}_{k}\right)\times \frac{{NDVI}_{i,m}}{\sum_{n=1}^{12}{NDVI}_{i,n}}$$

where *E*_rice,*i*,*k*,*m*_ represents the CH_4_ emissions (t); *A*_*i*,*k*_ denotes the cultivation area (km^2^) of rice type *k* (single- or double-cropping) in city *i*, obtained via zonal statistics from land cover data; EF_*k*_ is the annual emission factor, set as 236.7 kgCH_4_/ha for single-cropping rice and 241.0 kgCH_4_/ha for double-cropping rice; and the fractional term represents the temporal allocation coefficient, derived from the ratio of the aggregated NDVI value in month *m* (NDVI_*i*,*m*_) to the annual total NDVI sum for city *i*.

### Calculation of the CH_4_ Emissions from the Waste Sector

#### CH_4_ Emissions from Municipal Solid Waste Disposal

The mainstream estimation method adopted by the IPCC is the first-order decay (FOD) model, which has been widely used in 196 countries and regions under the United Nations Framework Convention on Climate Change (UNFCCC). The FOD model contains two key parameters: the waste recovery rate and the oxidation factor of CH_4_. Each landfill site selects the parameters from the default values based on on-site conditions to calculate the corresponding annual emissions. We assume that the basic physical and chemical properties of the same type of garbage in different landfill units do not show significant differences. We also assume that during the simulation, factors such as landfill operation mode, compaction density, cover layer type, leachate discharge and circulation conditions, and landfill gas collection efficiency will not undergo significant changes in time and space. We further assume that the key environmental factors affecting FOD degradation (temperature, moisture content, pH value) will not change significantly in time and space. Under the above unified assumption framework, we conduct the methane emission accounting for landfill sites.

This study primarily adopted the FOD method to estimate CH_4_ emissions from CHRED’s 1925 municipal solid waste landfills, presented in the following equation.

6 $$\begin{array}{l} E_{SW,i,y,m}=MSW_{SW,i,y,m}\times \sum_{q}^{4}(MCF\times f_{q}\times DOC_{q}\times DOC_{F}\times \\ \left(e^{-\left(T-1\right)\times k_{q}}-e^{-\left(T\times k_{q}\right)}\right))\times F\times \frac{16}{12}\times \left(1-R\right)\times \left(1-OX\right) \end{array}$$

where *E*_MS,*i*,*y*,*m*_ is CH_4_ emissions from solid waste disposal site *i* (g) in year *y* and month *m*; MSW means the amount of disposed waste (*t*), which is derived from the city’s statistical yearbook; MCF stands for the revised factor of CH_4_; *f*_*q*_ represents the fraction of different waste components, with *q* denoting waste categories (including food waste, paper, textiles, bamboo/wood, and others); DOC_*q*_ refers to the degradable organic carbon content; DOC_*F*_ is the fraction of decomposed DOC_*q*_; *T* is the length of time this landfill has been in operation at the time of this study; *k*_*q*_ is the reaction constant; *F* denotes the fraction of CH_4_ in landfill gas; *R* is the methane recovery rate; and OX represents the oxidation factor of CH_4_. The FOD emission factor data are based on studies.^25,40^ Detailed data are summarized in Tables S12–S14.

### CH_4_ Emissions from Sewage Treatment Plants

The estimation of CH_4_ emissions from CHRED’s 10,637 sewage treatment plants is presented according to the following equation.

7 $$E_{ST,i,y,m}=TWO_{ST,i,y,m}\times EF_{ST,i,y,m}-AT_{ST,i,y,m}$$

where *E*_ST,*i*,*y*,*m*_ is the amount of CH_4_ emissions generated from organic matter treatment; TOW_ST,*i*,*y*,*m*_ represents the total organics (in terms of degradable organic matter) in the influent; EF_ST,*i*,*y*,*m*_ is the methane emission factor; and AT_ST,*i*,*y*,*m*_ denotes the annual amount of CH_4_ recovered from anaerobic treatment. Notably, methane recovery is currently negligible in most wastewater treatment plants in China. We then estimated monthly sewage treatment volumes for each plant using provincial-level monthly sewage treatment statistics as proxies. Data on sewage treatment volumes were obtained from the China Environmental Statistical Yearbook and the Wind database.

### LMDI Decomposition

We applied the LMDI decomposition method to quantify the contributions of different factors to changes in city-level CH_4_ emissions. This method is widely used in energy and emission accounting because of its consistency, completeness, and lack of residual terms. In line with the Kaya identity, changes in methane emissions are attributed to demographic, economic, structural, and efficiency-related factors. Methane emissions are calculated as follows

8 $$M=\frac{\sum M_{i}}{M}\times \frac{M}{GDP}\times \frac{GDP}{POP}\times POP=S\times T\times E\times P$$

where *M*_*i*_ denotes emissions from sector *i* (energy activities, agricultural activities, and waste treatment), and

(1) *P* (population effect) represents population scale (total population),
(2) *E* (economic effect) represents economic level (gross domestic product, GDP per capita),
(3) *T* (emission-intensity effect) represents emission intensity per unit of economic output (CH_4_ emissions per unit GDP), and
(4) *S* (structural effect) represents the sectoral composition of emissions, defined as the distribution of emissions across energy activities, agriculture activities and waste treatment).

In the additive LMDI decomposition, sector-specific structural contributions were calculated for these three sectors and then summed to obtain the total structural effect. The sector-specific values are reported in the Results and figures as energy activities, agricultural activities, and waste treatment effects.

We summarized the data sources and preprocessing steps for all driving-force parameters used in the LMDI decomposition in Table S15. GDP was converted to constant 2018 prices using prefecture-level city-specific year-on-year price indices (relative to the previous year). The sectoral structural effect, corresponding to *S* in the LMDI decomposition, was calculated from the sectoral emission shares derived from our sector-resolved city-level CH_4_ inventory. The structural factor was calculated from our sector-resolved city-level CH_4_ inventory as sectoral emission shares. Sector definitions were kept consistent across years. For the logarithmic terms in LMDI, we checked for zero values across adjacent years. In our data set, zeros only occurred when a variable was zero in both adjacent years. In these cases, the corresponding contribution was set to zero. Missing-year values, if any, for socioeconomic drivers were carried forward using the previous year’s data (LOCF). When resident population was unavailable, registered population was used as a proxy. Changes in total emissions between a base years *t*_0_ and year *t* are decomposed additively as

9 $$\Delta M=M^{t}-M^{0}=\Delta S+\Delta T+\Delta E+\Delta P$$

where Δ*S*, Δ*T*, Δ*E* and Δ*P* denote the contributions of structural, emission-intensity, economic and population effects, respectively. Following the standard LMDI additive formulation, each component is calculated as

10 $$\begin{array}{l} \Delta S=\sum \frac{M^{t}-M^{0}}{\ln\left(M^{t}/M^{0}\right)}\times \ln\frac{S^{t}}{S^{0}}\\ \Delta T=\sum \frac{M^{t}-M^{0}}{\ln\left(M^{t}/M^{0}\right)}\times \ln\frac{T^{t}}{T^{0}}\\ \Delta E=\sum \frac{M^{t}-M^{0}}{\ln\left(M^{t}/M^{0}\right)}\times \ln\frac{E^{t}}{E^{0}}\\ \Delta P=\sum \frac{M^{t}-M^{0}}{\ln\left(M^{t}/M^{0}\right)}\times \ln\frac{P^{t}}{P^{0}} \end{array}$$

This formulation yields a full additive decomposition without residual terms, allowing us to distinguish between positive drivers (factors that increase methane emissions) and negative drivers (factors that offset emission growth). It therefore provides an interpretable and internally consistent basis for assessing the relative contributions of population, economic activity, structural change, and emission intensity to city-level methane emission changes.

### Methane Emission Scenario Prediction

For the sectoral prediction model, multiscale and long-term historical data from 2010 to 2024 were adopted for full-process calibration and effectiveness validation. Activity data was derived from continuous annual statistical yearbooks at national and local levels, while emission factors were referenced from national and provincial greenhouse gas inventory guidelines. The projection framework is developed based on the IPCC AR6 scenario pathways, constrained by China’s 2035 Nationally Determined Contribution (NDC) targets and 2060 carbon neutrality goal. Fully considering provincial differences in future energy demand, macroeconomic and demographic trends, sectoral activity levels, and the progress of emission reduction technologies (Tables S16–S26), this study adopts a widely recognized and peer-reviewed methodological framework^20,41,42^ to project China’s sectoral methane emissions.

#### Methane Emissions from the Energy Sector

In accordance with the global 1.5 °C target stipulated in the Paris Agreement and the national voluntary contribution (NDC) targets, based on the scenarios of IPCC AR6,^43^ and taking into account the heterogeneity of key driving factors such as urban population growth, increased consumption levels, energy structure transformation, and technological iteration and upgrading,^17^ the focus is on the methane emission sources of the two core energy activities—coal mining and oil and gas production. The emission factors are selected from the default emission factors of the IPCC and the recommended values in the Guidelines for the Compilation of Provincial Greenhouse Gas Inventories. An energy production process methane emission assessment model coupling the activity level and emission factors at the urban scale is constructed. The model is verified using historical emission measurement data from 2010 to 2024, and the evolution path of methane emissions in the urban energy production sector from 2025 to 2060 is systematically predicted.

#### Methane Emissions from the Agricultural Sector

This study was based on the core parameters related to CH_4_ emissions under the emission module of the Coupled Model Intercomparison Project Phase 6 (CMIP6) in the SSP database. Drawing on existing methods,^17,23^ the model incorporated key drivers related to population, economic development, production methods, and agricultural activities. Emission factors were selected from the IPCC default values and the recommended values in the Guidelines for the Compilation of Provincial Greenhouse Gas Inventories. The model was validated using historical emission data from 2010 to 2024 and used to predict agricultural CH_4_ emission pathways from 2025 to 2060.

#### Methane Emissions from the Waste Sector

Based on the IPCC AR6 scenario database, this study incorporates the core driving parameters of population, urbanization, and economic development under the SSP scenarios, to analyze the trend of changes in the proportion of municipal waste landfill and the amount of sewage treatment in the future. It uses the default factors of IPCC and the recommended emission factors recommended by Guidelines for the Compilation of Provincial Greenhouse Gas Inventories to calculate the methane emissions from the landfill and sewage treatment processes through the top-down method. After being verified by historical measured data from 2010 to 2024, the study systematically predicts the evolution path of methane emissions in the waste treatment field from 2025 to 2060.

### Uncertainty Analysis

Along with the IPCC’s uncertainty-analysis guidance, we employed the Monte Carlo simulation method to estimate uncertainty in CH_4_ accounting for Chinese cities, accounting for uncertainties in activity data and emission factors in the energy, agriculture, and waste disposal sectors. To achieve the uncertainty accounting of each sector, the specific implementation process is as follows. First, for the activity-level data and sector-specific emission factor parameters, normal probability distributions are assumed. Second, through 10,000 random samplings and iterative calculations, based on the activity levels and emission factor parameters obtained from the sampling, the methane emissions corresponding to the distribution are simulated. Finally, through statistical analysis, the 95% confidence interval of the methane emission estimates was determined, thereby characterizing the uncertainty range of the emission accounting results. The detailed parameters for sectoral probability distribution functions were determined based on the completeness of city-level officially published energy data (as shown in Table S2) and our previous study on the city-level GHG emission inventory.^44^

## Supplementary Material



## Notes

Additional benchmarking against existing inventories, regional sectoral decomposition, lists of representative cities, supplementary figures for projected sector-specific reductions, data sources for activity levels and emission factors, FOD model parameters, LMDI parameter definitions, and staged methane mitigation pathway assumptions (PDF)
