# Supplementary material for: City-Scale, Source-Resolved Methane Inventories Reveal Drivers and Mitigation Pathways Across China’s Cities
Source: Environ Sci Technol. 2026 Aug 3;60(32):22323–34. doi: 10.1021/acs.est.5c18654 (PMC13492280; doi:10.1021/acs.est.5c18654)
Supplement: Supplementary file 1 [file es5c18654_si_001.pdf]

Supplementary Materials

City-scale, source-resolved methane inventories reveal drivers and mitigation pathways across China's cities

Li Zhang[1,2,#], Yifan Chen[3,#], Ke Wang[4,#], Jing Guo[5,*], Sen Liang[6], Pengchen Wu[7], Haoyu Zhang[8], Jingyi Wu[9], Ying Cui[10], Chen Lv[11], Hui Xu[2], Qunwei Wang[11], Bofeng Cai[5], Jinnan Wang[5], Linyan Li[3,12,*]

[1] School of the Environment and Safety Engineering, Jiangsu University, Zhenjiang, 212013, China
[2] Jiangxi Provincial Key Laboratory of Greenhouse Gas Accounting and Carbon Reduction, Institute of Energy Research, Jiangxi Academy of Sciences, Nanchang, 330096, China.
[3] Department of Data Science, City University of Hong Kong, Hong Kong SAR 999077, China
[4] School of Land Science and Technology, China University of Geosciences, Beijing 100083, China
[5] Center for Carbon Neutrality, Chinese Academy of Environmental Planning, Beijing, 100043, China
[6] Chinese Academy of Natural Resources Economics, Sanhe City, 101149, China
[7] School of Public Health, Sun Yat-sen University, Guangzhou, Guangdong 510080, China
[8] State Grid Shaanxi Electric Power Company Limited Research Institute, Xi'an, 710065, China
[9] School of Environmental Science and Engineering, Shanghai Jiao Tong University, Shanghai Engineering Research Center of Solid Waste Treatment and Resource, Shanghai 200240, China
[10] School of Environmental and Chemical Engineering, Shanghai University of Electric Power, Shanghai 200090, China
[11] College of Economics and Management, Nanjing University of Aeronautics and Astronautics, Nanjing 211106, China.
[12] Department of Infectious Diseases and Public Health, City University of Hong Kong, Hong Kong SAR 999077, China
#These authors contributed equally to this work.
*Correspondence to: Jing Guo (guojing@caep.org.cn) and Linyan Li (linyanli@cityu.edu.hk)

SI Section S1. Comparison with existing inventories and studies

Standardization of the full model calibration and validation workflow: First, core parameters including sectoral emission factors, activity level elastic coefficients, and emission reduction technology diffusion curves were calibrated using national and provincial historical data from 2010 to 2020. Subsequently, the period 2021–2024 was selected as the out-of-sample validation interval. Model simulated emissions were cross-compared and verified with official national inventories released by the Ministry of Ecology and Environment of China, self-compiled provincial inventories, and mainstream authoritative global datasets.

We benchmarked our city-based CH_4 inventory aggregated to national and provincial totals against representative existing inventories for overlapping years (Tables S4–S6). At the national level, our total anthropogenic CH_4 emissions are consistently higher than EDGAR[1] by about 14%–17% during 2018–2024 and higher than MMCP[2] by about 19%–25% during 2018–2022 (Table S4). The sectoral structure is broadly comparable across datasets, while the largest absolute differences are concentrated in the energy sector. EDGAR reports higher waste emissions than this study in earlier years, and the waste estimates converge in recent years (Table S4).

At the provincial level, the 2022 totals aggregated from our 339-city inventory show strong consistency with both EDGAR and MMCP, as indicated by high Pearson correlations ($r = 0.906$ for EDGAR and $r = 0.914$ for MMCP; Table S5). Province-by-sector comparisons against MMCP further show that agreement is highest for agriculture, followed by waste, while energy exhibits substantially larger deviations (Table S6). The median absolute relative differences are 10% for agriculture and 25% for waste, compared with 53% for energy. These differences are

expected because energy-related CH_4, especially from coal mining and oil and gas systems, is sensitive to emission-factor choices, activity statistics, and spatial allocation methods that vary across inventories.

Overall, the cross-inventory comparison supports the robustness of our national magnitude and provincial spatial patterns in overlapping years. The remaining discrepancies are explainable by differences in system boundaries, sector definitions and mapping rules, and emission factors and activity data used by each inventory. These differences do not affect the main conclusions regarding city-level heterogeneity, driver attribution, and mitigation scenarios.

SI Section S2. Drivers of differences across inventories

Differences across inventories mainly arise from four aspects. First, activity-data sources and resolution differ, including how energy production, livestock, and waste-treatment activities are compiled and harmonized across administrative levels. Second, emission factors and key parameters vary across inventories, especially for coal mining and oil and gas systems, manure management, landfill decay, and wastewater pathways, which can shift sector totals. Third, system boundaries and sector coverage are not fully consistent, such as anthropogenic-only definitions and whether specific subsectors are included. Fourth, spatial allocation approaches differ when downscaling emissions, which affects provincial and city aggregates and can lead to localized discrepancies even when national totals are comparable.

SI Section S3. Assumptions for staged methane mitigation pathways in the scenario analysis

Future methane emission pathways were developed based on population and economic

projections from the Shared Socioeconomic Pathways (SSP) database, together with anticipated changes in China's energy structure, the expansion of non-fossil energy capacity and consumption, and transitions in waste treatment practices. On this basis, we characterized the likely evolution of activity levels in the energy, agriculture, and waste sectors and further incorporated expected improvements in methane mitigation technologies within each sector. These assumptions were constrained by the IPCC AR6 climate scenarios, China's nationally determined contribution targets, and the long-term carbon neutrality goal. Following the accounting framework used in this study, the scenario analysis projects a continued decline in China's methane emissions after 2025. Relative to 2025, the average annual growth rates were estimated at -3.7% for 2025–2030 and -1.3% for 2031–2035, corresponding to an overall reduction of about 15%–20% by 2035 relative to the peak level. In terms of mitigation contributions, energy transition and technological strengthening measures accounted for approximately 62.9% and 37.1% of the total reduction, respectively, with coal mining and solid waste treatment together contributing about 77%. Based on existing literature[3–8], we further summarized the representative methane mitigation pathways for three periods: 2025–2030, 2031–2035, and 2036–2060 (Table S27).

2025–2030

This period is characterized by the implementation of mandatory control policies and the establishment of monitoring, reporting, and verification (MRV) capacity. Technology deployment is expected to focus on low-cost, high-efficiency recovery measures and more precise identification of methane sources.

In the energy sector, the main priorities are low-concentration methane utilization and leakage control in coal mining, as well as associated gas recovery and leak detection and repair

in oil and gas systems.

In the agricultural sector, alternate wetting and drying irrigation is expected to be further promoted in rice cultivation. In livestock production, low-protein feed formulations and methane inhibitors such as 3-NOP are expected to enter early-stage application. At the same time, biogas projects for livestock and poultry manure management are expected to expand further, capturing methane through anaerobic digestion and converting it into usable energy.

In the waste sector, ongoing “zero-waste city” initiatives are expected to accelerate the transition of municipal solid waste treatment from landfill-dominated systems to incineration-based systems. For existing landfills, landfill gas collection and energy recovery technologies are expected to be more widely adopted. In wastewater treatment, methane control is expected to increasingly rely on anaerobic digestion of sludge, allowing methane generated during treatment to be recovered and utilized.

2031–2035

This period is characterized by more refined, integrated, and value-added mitigation strategies. Policy emphasis is expected to shift from basic methane recovery and utilization toward whole-process collaborative management.

In the energy sector, priority is expected to move toward integrated ground-based and remote sensing monitoring systems, together with methane control in closed and abandoned mines. As outdated coal production capacity is phased out, methane extraction from abandoned mines is expected to become increasingly important for avoiding uncontrolled post-closure emissions. In the oil and gas sector, low-emission green well completion technologies and digital platforms for leak detection and repair (LDAR) are expected to be deployed more broadly across the value chain.

In the agricultural sector, methane mitigation in rice systems is expected to combine water management with soil improvement measures such as biochar application. In livestock systems, breeding programs for low-methane-emission animals are expected to make further progress. For manure management, larger-scale centralized biogas systems, together with improved utilization of digestate and biogas slurry, are expected to support more integrated crop-livestock systems.

In the waste sector, the technical focus is expected to shift toward efficient anaerobic digestion and more separate treatment of organic waste streams, improving methane recovery and reducing uncontrolled emissions.

2036–2060

This period is characterized by the management of residual emissions and the increasing role of near-zero and negative-emission approaches under a substantially decarbonized energy system. The long-term goal is near-zero methane emissions across the energy, agriculture, and waste sectors.

In the energy sector, conventional methane venting is expected to be largely eliminated, and control efforts are expected to focus on residual leakage from closed mines and remaining fossil fuel infrastructure. Bioremediation and ultra-low-concentration methane oxidation technologies may play a growing role in addressing diffuse emissions from abandoned mines. Oil and gas extraction activities are expected to decline substantially, while the remaining facilities move toward near-zero leakage. Monitoring systems are also expected to rely increasingly on automated remote sensing and high-resolution satellite verification.

In the agricultural sector, supply-side mitigation may increasingly depend on advanced breeding and biotechnology approaches, including gene editing for low-emission, high-yield rice and livestock varieties. On the demand side, the large-scale adoption of alternative proteins,

including plant-based and cell-cultured products, may reduce demand for ruminant meat and thereby lower methane emissions from enteric fermentation.

In the waste sector, under a near "zero landfill" system, integrated treatment of mixed organic waste is expected to support greater production of biogas and organic fertilizers. Waste management may also be increasingly combined with biological carbon sequestration and other negative-emission approaches to further reduce residual carbon and methane emissions.

Table S1. Comparative analysis of methane emissions in this study and official national emission inventory

Year	Official national emission inventory (10^4t)	This study (10^4t)	Absolute difference (%)
2018	6013.2	6012.6	0.010%
2019	/	5957.3	/
2020	5902.8	6048.2	2.463%
2021	5953.5	5952.9	0.010%
2022	/	5991.2	/
2023	/	6042.3	/
2024	/	6169.4	/

Table S2. Summary of activity-data, emission-factor, and combined emission uncertainties for the energy, agriculture, and waste sectors in this study

Sectors	Activity data uncertainty	Emission factor uncertainty	Emission uncertainty
**Energy sector**			
Coal mining	20%	30%	21%
Oil and gas extraction	15%	10%	12%
**Agriculture sector**			
Enteric fermentation & Manure management	8%	30%	15%
Rice cultivation	20%	10%	14%
**Waste treatment sector**			
Landfill	20%	20%	20%
Wastewater treatment	20%	25%	22%

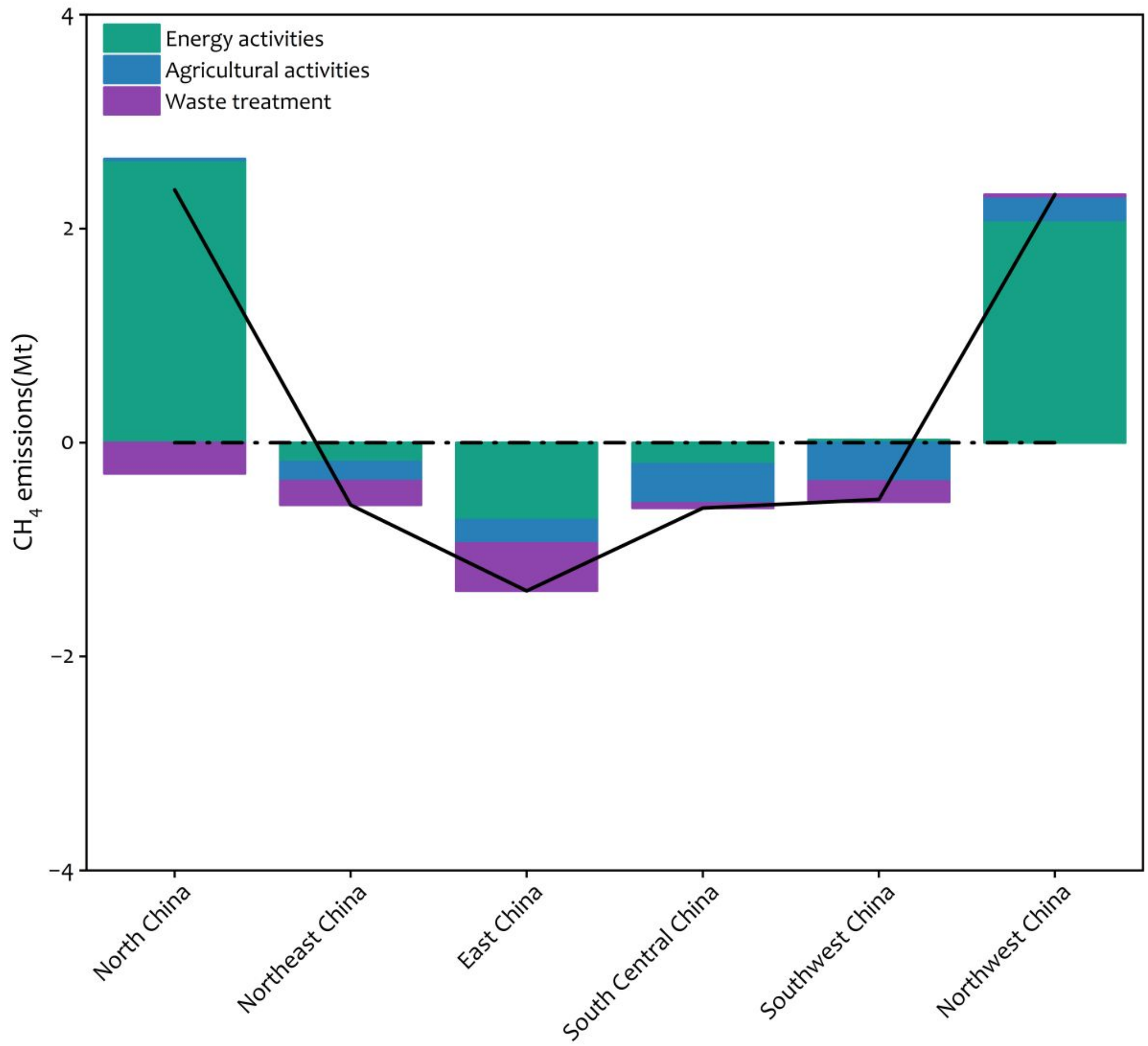

Fig. S1 | Regional sectoral decomposition of methane emission changes in China (2018–2024). Bars show sectoral contributions from energy activities, agricultural activities, and waste treatment to the net regional change.

Table S3. Regional methane emissions in 2018 and 2024 and sectoral contributions to changes (2018–2024). Units: kt CH_4 yr^{-1}

**Region**	**Emissions (2018)**	**Emissions (2024)**	**Change (2018–2024)**	**Change (%)**	**Energy: change**	**Energy: share (%)**	**Agriculture: change**	**Agriculture: Share (%)**	**Waste: change**	**Waste: share (%)**
North China	18716	21078	+2362	+12.6%	+2641	111.8	+12	0.5%	-290	-12.3%
Northeast China	5392	4808	-584	-10.8%	-181	31.1	-180	30.8%	-223	38.2%
East China	9003	7617	-1386	-15.4%	-723	52.1	-220	15.9%	-442	31.9%
South Central China	8544	7931	-613	-7.2%	-202	33.0	-365	59.5%	-46	7.5%
Southwest China	7667	7136	-531	-6.9%	+24	-4.5	-361	68.1%	-193	36.5%
Northwest China	10805	13123	+2318	+21.5%	+2080	89.8	220	9.5%	17	0.7%
China (total)	60127	61693	+1566	2.6%	+3639	232.4	-894	-57.1%	-1177	-75.2%

Table S4. Benchmarking of national CH_4 emissions and sectoral breakdowns against existing inventories (overlapping years).

Year	This study (Mt)				EDGAR						MMCP					
	Total (Mt)	Energy	Agriculture	Waste	Total (Mt)	Energy	Agricultur e	Waste	ΔTotal (EDGAR–This)	%ΔTotal	Total (Mt)	Energy	Agricultur e	Waste	ΔTotal (MMCP–This)	%ΔTotal
2018	60.13	28.66	23.85	7.62	50.38	22.12	17.10	11.16	-9.74	-16.0%	45.25	14.02	22.52	8.71	-14.88	-25.0%
2019	59.57	28.29	23.78	7.50	50.26	22.70	16.63	10.93	-9.32	-16.0%	45.98	14.59	22.41	8.98	-13.60	-23.0%
2020	60.48	29.38	23.72	7.38	50.44	22.92	16.99	10.54	-10.04	-17.0%	46.65	14.97	22.24	9.45	-13.83	-23.0%
2021	59.53	28.17	24.28	7.08	51.02	24.02	17.15	9.85	-8.51	-14.0%	47.62	15.64	22.69	9.30	-11.91	-20.0%
2022	59.91	30.58	22.51	6.82	50.60	25.13	17.04	8.43	-9.31	-16.0%	48.78	16.90	22.06	9.82	-11.13	-19.0%
2023	60.42	31.28	22.58	6.56	51.07	25.80	17.87	7.40	-9.36	-15.0%	-	-	-	-	-	-
2024	61.69	32.30	22.95	6.44	50.92	26.11	17.82	6.99	-10.78	-17.0%	-	-	-	-	-	-

Table S5. Benchmarking of province-level CH_4 totals against existing inventories (2022).

Province	This study total (kt)	Inventory EDGAR total (kt)	Δ (EDGAR–This)	%Δ	Inventory MMCP total (kt)	Δ (MPCC–This)	%Δ
Beijing	248.82	239.55	-9.27	-4.0%	316.55	67.72	27.0%
Tianjin	220.41	217.48	-2.93	-1.0%	368.13	147.72	67.0%
Hebei	1416.48	2797.77	1381.28	98.0%	1358.85	-57.63	-4.0%
Shanxi	12174.70	7695.34	-4479.36	-37.0%	5922.29	-6252.41	-51.0%
Inner Mongolia	6799.62	7683.62	884.00	13.0%	3086.93	-3712.69	-55.0%
Liaoning	1197.24	1078.98	-118.26	-10.0%	1170.58	-26.66	-2.0%
Jilin	918.38	565.09	-353.28	-38.0%	731.59	-186.78	-20.0%
Heilongjiang	2675.06	1099.91	-1575.15	-59.0%	1787.62	-887.44	-33.0%
Shanghai	259.49	445.55	186.07	72.0%	374.69	115.20	44.0%
Jiangsu	1118.05	2029.90	911.85	82.0%	1538.73	420.69	38.0%
Zhejiang	527.23	1066.66	539.43	102.0%	708.79	181.56	34.0%
Anhui	2197.99	2777.26	579.27	26.0%	1876.86	-321.13	-15.0%
Fujian	493.22	593.48	100.26	20.0%	544.28	51.06	10.0%
Jiangxi	1174.74	1108.93	-65.81	-6.0%	1420.01	245.28	21.0%
Shandong	1679.06	2358.98	679.91	40.0%	1447.19	-231.87	-14.0%
Henan	2235.37	1758.05	-477.32	-21.0%	2060.22	-175.15	-8.0%
Hubei	1301.35	1305.72	4.38	0.0%	1752.43	451.09	35.0%
Hunan	1845.23	1476.18	-369.05	-20.0%	2194.24	349.01	19.0%
Guangdong	1215.03	1561.46	346.44	29.0%	2487.74	1272.72	105.0%
Guangxi	1002.09	1012.64	10.55	1.0%	1420.54	418.45	42.0%
Hainan	218.63	155.08	-63.55	-29.0%	261.11	42.48	19.0%
Chongqing	514.75	429.97	-84.77	-16.0%	732.50	217.76	42.0%
Sichuan	2375.23	1370.19	-1005.04	-42.0%	2767.99	392.76	17.0%
Guizhou	1639.69	759.66	-880.04	-54.0%	2113.28	473.59	29.0%
Yunnan	1668.15	675.55	-992.60	-60.0%	1797.52	129.37	8.0%
Tibet	682.03	180.74	-501.29	-73.0%	518.31	-163.73	-24.0%
Shaanxi	5358.40	4316.57	-1041.83	-19.0%	3097.34	-2261.06	-42.0%

Gansu	1262.42	784.60	-477.81	-38.0%	953.69	-308.73	-24.0%
Qinghai	857.20	252.74	-604.46	-71.0%	851.45	-5.75	-1.0%
Ningxia	1077.94	1154.94	77.00	7.0%	786.08	-291.86	-27.0%
Xinjiang	3558.47	1650.75	-1907.72	-54.0%	2330.16	-1228.31	-35.0%

Table S6. Benchmarking of province-by-sector CH_4 emissions against an existing inventory (2022).

Province	This study Energy	MMCP Energy	%Δ	This study Agriculture	MMCP Agriculture	%Δ	This study Waste	MMCP Waste	%Δ
Beijing	0.45	9.32	1975.0%	13.14	10.18	-22.0%	235.24	297.05	26.0%
Tianjin	124.55	167.24	34.0%	39.98	58.95	47.0%	55.88	141.94	154.0%
Hebei	437.60	207.47	-53.0%	642.75	740.58	15.0%	336.13	410.81	22.0%
Shanxi	11625.24	5460.48	-53.0%	287.26	234.71	-18.0%	262.20	227.10	-13.0%
Inner Mongolia	5150.99	1586.19	-69.0%	1352.83	1272.56	-6.0%	295.80	228.18	-23.0%
Liaoning	292.75	173.07	-41.0%	508.84	507.30	0.0%	395.65	490.21	24.0%
Jilin	73.75	87.77	19.0%	665.15	460.62	-31.0%	179.48	183.21	2.0%
Heilongjiang	502.19	468.44	-7.0%	1933.55	1056.10	-45.0%	239.32	263.08	10.0%
Shanghai	12.46	19.04	53.0%	34.51	37.81	10.0%	212.51	317.84	50.0%
Jiangsu	108.90	49.38	-55.0%	812.90	867.02	7.0%	196.25	622.33	217.0%
Zhejiang	11.12	8.03	-28.0%	231.20	217.57	-6.0%	284.91	483.18	70.0%
Anhui	979.04	516.13	-47.0%	994.35	1093.89	10.0%	224.60	266.84	19.0%
Fujian	7.09	33.89	378.0%	265.94	241.09	-9.0%	220.20	269.30	22.0%
Jiangxi	4.58	30.96	575.0%	948.60	1154.79	22.0%	221.55	234.26	6.0%
Shandong	656.47	290.48	-56.0%	705.89	590.49	-16.0%	316.71	566.22	79.0%
Henan	737.33	375.74	-49.0%	1136.28	1073.83	-5.0%	361.75	610.66	69.0%
Hubei	44.92	35.96	-20.0%	1088.84	1333.27	22.0%	167.58	383.21	129.0%
Hunan	18.46	70.85	284.0%	1526.10	1700.59	11.0%	300.67	422.79	41.0%
Guangdong	66.01	203.99	209.0%	547.26	1049.96	92.0%	601.76	1233.80	105.0%
Guangxi	9.52	50.38	429.0%	806.91	1138.01	41.0%	185.65	232.16	25.0%
Hainan	49.50	37.21	-25.0%	131.91	163.16	24.0%	37.22	60.74	63.0%
Chongqing	0.14	104.09	73589.0%	388.64	372.86	-4.0%	125.97	255.56	103.0%
Sichuan	323.80	706.75	118.0%	1721.24	1627.75	-5.0%	330.19	433.50	31.0%
Guizhou	786.46	1281.18	63.0%	707.66	651.87	-8.0%	145.57	180.24	24.0%
Yunnan	253.34	422.31	67.0%	1235.93	1188.43	-4.0%	178.88	186.78	4.0%
Tibet	0.03	0.09	173.0%	653.99	487.98	-25.0%	28.01	30.24	8.0%
Shaanxi	4822.59	2497.07	-48.0%	276.46	266.57	-4.0%	259.35	333.71	29.0%

Gansu	459.91	172.25	-63.0%	692.47	651.22	-6.0%	110.04	130.22	18.0%
Qinghai	117.23	81.03	-31.0%	676.17	713.38	6.0%	63.80	57.04	-11.0%
Ningxia	755.58	450.69	-40.0%	292.05	276.49	-5.0%	30.31	58.90	94.0%
Xinjiang	2147.97	1305.96	-39.0%	1193.68	819.62	-31.0%	216.82	204.58	-6.0%

Table S7. Cities where energy accounts for > 50% of total CH_4 emissions (2024)

City	Region	Total CH_4 2024 (kt)	Energy share 2024 (%)
Jincheng	North China	1793.8	99%
Yangquan	North China	913.7	99%
Ordos	North China	4613.6	99%
Changzhi	North China	1676.3	99%
Wuhai	North China	263.5	97%
Yulin	Northwest China	3618.4	97%
Xianyang	Northwest China	936.9	97%
Shuozhou	North China	1634.4	96%
Lvliang	North China	1548.2	96%
Hami	Northwest China	738.8	95%
Jinzhong	North China	1201.2	95%
Huainan	East China	373.8	95%
Huaibei	East China	149.2	94%
Linfen	North China	865.0	92%
Liupanshui	Southwest China	566.5	91%
Tongchuan	Northwest China	127.4	91%
Datong	North China	757.8	90%
Xinzhou	North China	907.6	89%
Hulunbuir	North China	512.1	88%
Yinchuan	Northwest China	667.3	87%
Sanmenxia	South Central China	61.1	86%
Changji	Northwest China	807.1	85%
Taiyuan	North China	584.4	84%
Karamay	Northwest China	87.3	83%
Deyang	Southwest China	284.1	82%
Jining	East China	456.2	81%
Turpan	Northwest China	90.9	80%
Pingliang	Northwest China	274.0	79%
Daxing'anling	Northeast China	3.2	79%
Baoji	Northwest China	224.7	78%
Ili	Northwest China	325.2	78%
Bayingolin	Northwest China	423.5	77%
Yan'an	Northwest China	445.2	77%
Weinan	Northwest China	211.5	75%
Handan	North China	236.3	73%
Zaozhuang	East China	87.7	73%
Fuyang	East China	238.6	71%
Bijie	Southwest China	560.8	71%
Urumqi	Northwest China	245.3	71%
Qitaihe	Northeast China	61.9	71%
Baiyin	Northwest China	133.4	70%

Xuchang	South Central China	82.1	70%
Haikou	South Central China	80.4	70%
Suzhou	East China	116.9	68%
Shangqiu	South Central China	198.0	64%
Tieling	Northeast China	134.5	64%
Wuzhong	Northwest China	281.0	64%
Haixi	Northwest China	140.3	61%
Liaoyang	Northeast China	63.8	60%
Yuncheng	North China	111.0	60%
Pingdingshan	South Central China	296.6	60%
Hebi	South Central China	58.9	60%
Xinxiang	South Central China	110.4	57%
Xingtai	North China	115.4	57%
Puyang	South Central China	92.2	55%
Fushun	Northeast China	48.8	54%
Tai'an	East China	93.6	54%
Shizuishan	Northwest China	63.1	53%
Tangshan	North China	283.2	53%
Qianxinan	Southwest China	180.1	53%
Heze	East China	186.3	52%
Tianjin	North China	262.2	52%
Qujing	Southwest China	293.6	52%
Jiaozuo	South Central China	71.6	52%
Aksu	Northwest China	328.2	51%

170

Table S8. Cities where waste dominates total CH_4 emissions (2024)

City	Region	Total CH_4 2024 (kt)	Waste share 2024 (%)
Shenzhen	South Central China	103.6	99%
Dongguan	South Central China	43.2	99%
Zhongshan	South Central China	35.2	99%
Foshan	South Central China	65.6	97%
Shantou	South Central China	87.2	93%
Zhuhai	South Central China	19.8	91%
Beijing	North China	200.4	91%
Xiamen	East China	29.2	91%
Huizhou	South Central China	122.2	85%
Sanya	South Central China	10.2	84%
Guangzhou	South Central China	179.9	81%
Zhoushan	East China	6.3	80%
Ningbo	East China	77.3	78%
Chaozhou	South Central China	17.7	77%
Shanghai	East China	209.1	77%
Huludao	Northeast China	20.4	73%
Suzhou	East China	86.2	72%
Jiayuguan	Northwest China	4.8	72%
Xi’an	Northwest China	48.8	69%
Henan Jurisdictional County	South Central China	5.4	69%
Liaoyuan	Northeast China	21.7	68%
Wuxi	East China	40.1	66%
Nanjing	East China	94.2	66%
Hangzhou	East China	144.7	66%
Wuhan	South Central China	155.7	65%
Taizhou	East China	44.3	63%
Weihai	East China	16.4	62%
Shaoxing	East China	59.7	61%
Qingyuan	South Central China	126.2	60%
Chengdu	Southwest China	133.7	60%
Anshan	Northeast China	43.2	59%
Jinhua	East China	98.0	59%
Jieyang	South Central China	28.9	55%
Jiangmen	South Central China	75.9	55%
Qingdao	East China	80.6	51%
Quanzhou	East China	50.7	51%
Changzhou	East China	40.2	50%
Guiyang	Southwest China	53.7	50%

173

Table S9. Cities with the largest increases and decreases in CH_4 (2018–2024)

City	Region	CH_4 2018 (kt)	CH_4 2024 (kt)	Net change (kt)
Jining	East China	716.8	456.2	-260.6
Shanghai	East China	368.7	209.1	-159.6
Handan	North China	368.9	236.3	-132.6
Huainan	East China	501.4	373.8	-127.7
Beijing	North China	320.1	200.4	-119.7
Shenyang	Northeast China	386.7	275.4	-111.2
Huaibei	East China	257.2	149.2	-108.0
Jiamusi	Northeast China	452.2	351.5	-100.6
Urumqi	Northwest China	143.1	245.3	102.2
Taiyuan	North China	481.5	584.4	103.0
Yangquan	North China	804.6	913.7	109.1
Aksu	Northwest China	218.8	328.2	109.4
Qingyang	Northwest China	147.5	265.1	117.6
Yinchuan	Northwest China	549.6	667.3	117.7
Datong	North China	636.0	757.8	121.8
Linfen	North China	705.5	865.0	159.5
Jinzhong	North China	1013.9	1201.2	187.3
Ili	Northwest China	131.6	325.2	193.5
Shuozhou	North China	1416.1	1634.4	218.2
Xinzhou	North China	688.0	907.6	219.6
Changzhi	North China	1417.0	1676.3	259.3
Jincheng	North China	1517.3	1793.8	276.5
Changji	Northwest China	448.3	807.1	358.8
Yulin	Northwest China	3258.8	3618.4	359.6
Lvliang	North China	1071.0	1548.2	477.2
Hami	Northwest China	255.2	738.8	483.6
Ordos	North China	3977.5	4613.6	636.1

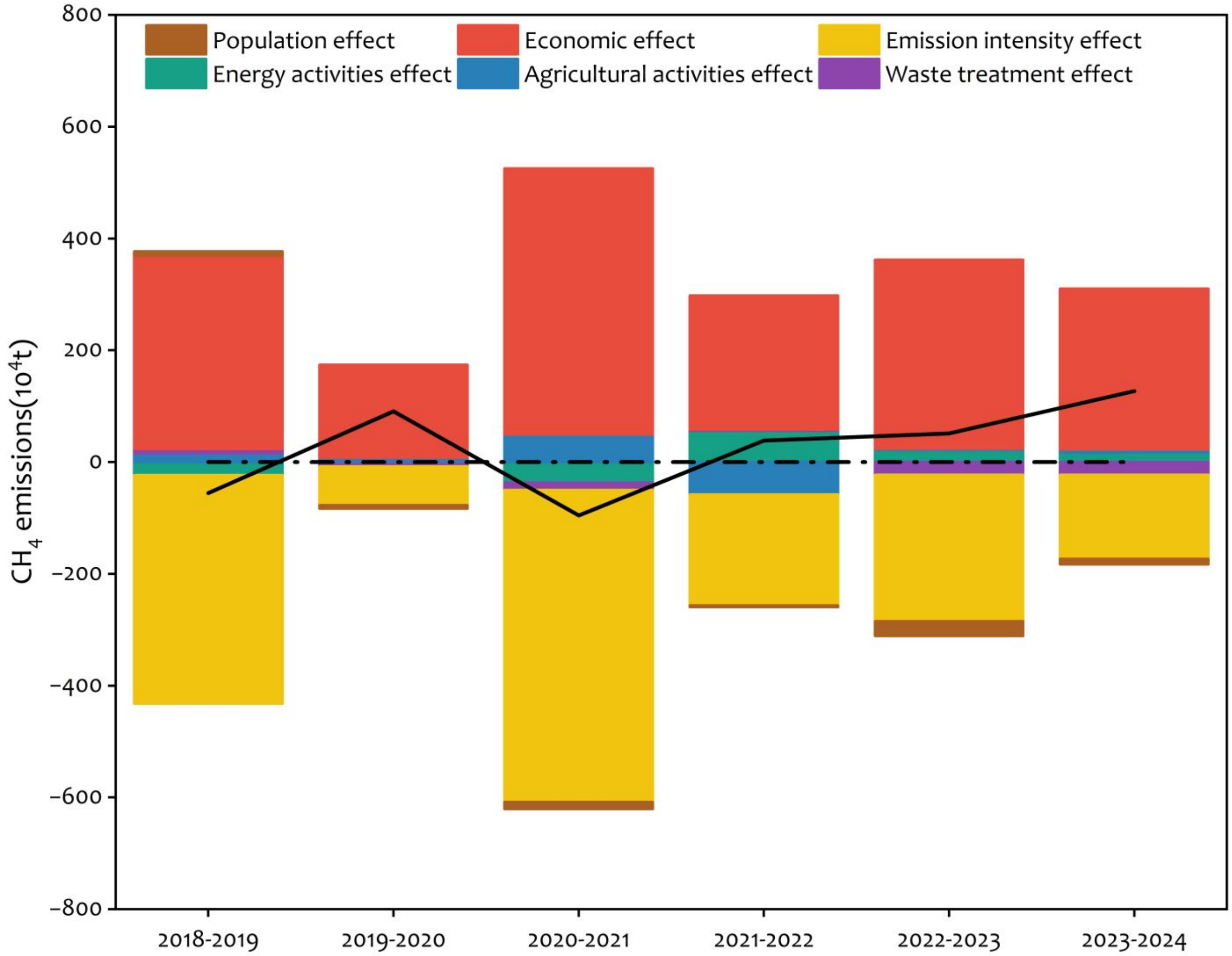

Fig. S2 | Decomposition of year-to-year changes in national CH_4 emissions in China (2018–2024).

Stacked bars show the contributions of the population effect, economic effect, emission intensity effect, and three sectoral structural components, including energy activities, agricultural activities and waste treatment, to changes in national CH_4 emissions between consecutive years (2018–2019 to 2023–2024). Positive contributions are plotted upward and negative contributions downward. The solid black line represents the net change in emissions (sum of all effects) for each interval, and the dashed horizontal line denotes zero change.

Table S10. City screening criteria and included cities for Fig. 3 by region

Region	Cutoff (kt)	Inclusion rule for Fig. 3a–f	Cities shown in Fig. 3a–f (list)	Selected city example in Fig. 3g	Notes
North China	100	Include if |total effect| > 100 or if any of six driver contributions has |value| > 100	Ordos, Lvliang, Jincheng, Changzhi, Xinzhou, Shuozhou, Jinzhong, Linfen, Datong, Yangquan, Taiyuan, Hulunbuir, Tongliao, Chifeng, Tangshan, Beijing, Handan	Jincheng	Threshold used only for figure display
Northwest China	100	Include if |total effect| > 100 or if any of six driver contributions has |value| > 100	Hami, Yulin, Changji, Ili, Yinchuan, Qingyang, Aksu, Urumqi, Bayingolin, Tacheng, Pingliang, Xianyang, Yan'an	Changji	Same rule as above
Northeast China	30	Include if |total effect| > 30 or if any of six driver contributions has |value| > 30	Harbin, Dalian, Songyuan, Baicheng, Daqing, Siping, Jilin, Suihua, Panjin, Mudanjiang, Qiqihar, Yanbian, Hegang, Tieling, Changchun, Jixi, Shuangyashan, Jiamusi, Shenyang	Shenyang	Same rule as above
Southwest China	40	Include if |total effect| > 40 or if any of six driver contributions has |value| > 40	Bijie, Qujing, Deyang, Qianxinan, Chuxiong, Suining, Zhaotong, Nagqu, Dali, Lijiang, Wenshan, Baoshan, Honghe, Tongren, Qamdo, Qiandongnan, Ziyang, Lhasa, Nanchong, Leshan, Guang'an, Luzhou, Mianyang, Ganzi, Rikaze, Liangshan, Aba, Guiyang, Zunyi, Dazhou, Chengdu, Chongqing, Liupanshui	Chongqing	Same rule as above
East China	50	Include if |total effect| > 50 or if any of six driver contributions has |value| > 50	Nanjing, Hefei, Lu'an, Yancheng, Ji'an, Shangrao, Hangzhou, Huai'an, Ganzhou, Bozhou, Chuzhou, Sanming, Fuzhou, Dezhou, Dongying, Qingdao, Tai'an,	Jining	Same rule as above

<table>
<tr><td></td><td></td><td></td><td>Linyi, Bengbu, Jinan, Yichun, Heze, Yantai, Suzhou, Fuyang, Xuzhou, Huaibei, Huainan, Shanghai, Jining</td><td></td><td></td></tr>
<tr><td>South Central China</td><td>50</td><td>Include if |total effect| > 50 or if any of six driver contributions has |value| > 50</td><td>Huizhou, Wuhan, Qingyuan, Shantou, Shaoyang, Huanggang, Hengyang, Nanning, Yueyang, Xiangyang, Hubei JC, Changde, Jingmen, Zhuzhou, Changsha, Jingzhou, Huaihua, Chenzhou, Luoyang, Xinxiang, Xiangtan, Xiaogan, Zhengzhou, Guilin, Zhumadian, Shenzhen, Guangzhou, Nanyang, Pingdingshan, Shangqiu</td><td>Changsha</td><td>Same rule as above</td></tr>
</table>

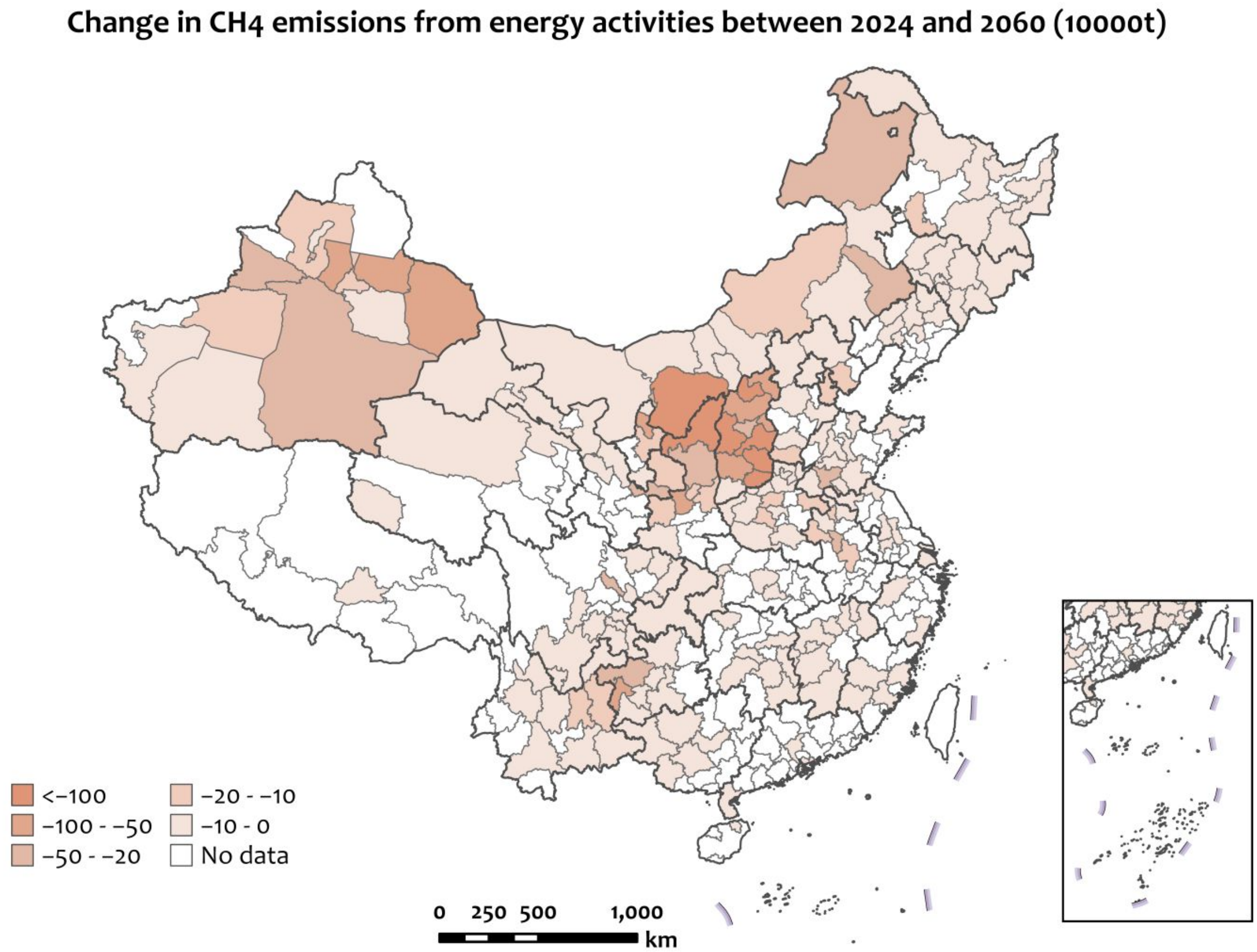

Fig. S3 | Projected changes in CH_4 emissions from energy activities between 2024 and 2060 in Chinese prefecture-level cities.

Map showing city-level changes in methane emissions from energy-related activities (coal, oil and gas) between 2024 and 2060, expressed in 10,000 t (10^4 t). Shading denotes the magnitude of emission reductions, from small decreases (−10 to 0 $\times 10^4$ t) to very large declines (<−100 $\times 10^4$ t), while white areas indicate cities with no data. Reductions are concentrated in key energy-producing regions in North and Northwest China, with more modest declines across much of the rest of the country.

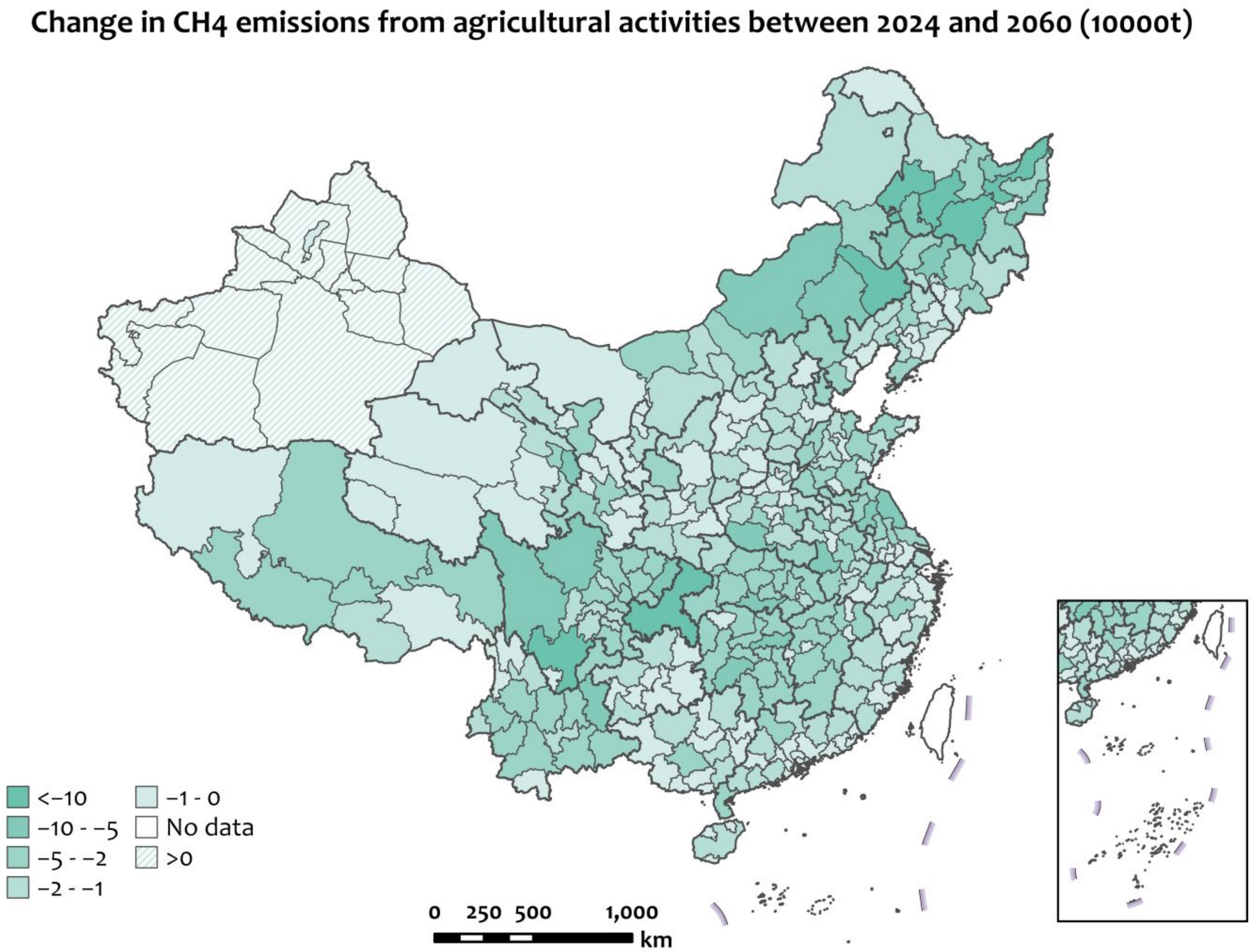

Fig. S4 | Projected changes in CH_4 emissions from agricultural activities between 2024 and 2060 in Chinese prefecture-level cities.

Map showing city-level changes in methane emissions from agricultural activities (enteric fermentation, manure management and rice cultivation) between 2024 and 2060, expressed in 10,000 t (10^4 t). Green shades indicate emission reductions of different magnitudes (from −1 to 0 $\times 10^4$ t to <−10 $\times 10^4$ t), while hatched areas mark cities with projected emission increases (>0 $\times 10^4$ t) and white areas denote cities with no data. Reductions are widespread in southern and central China, whereas several northern and western cities show limited increases or little change.

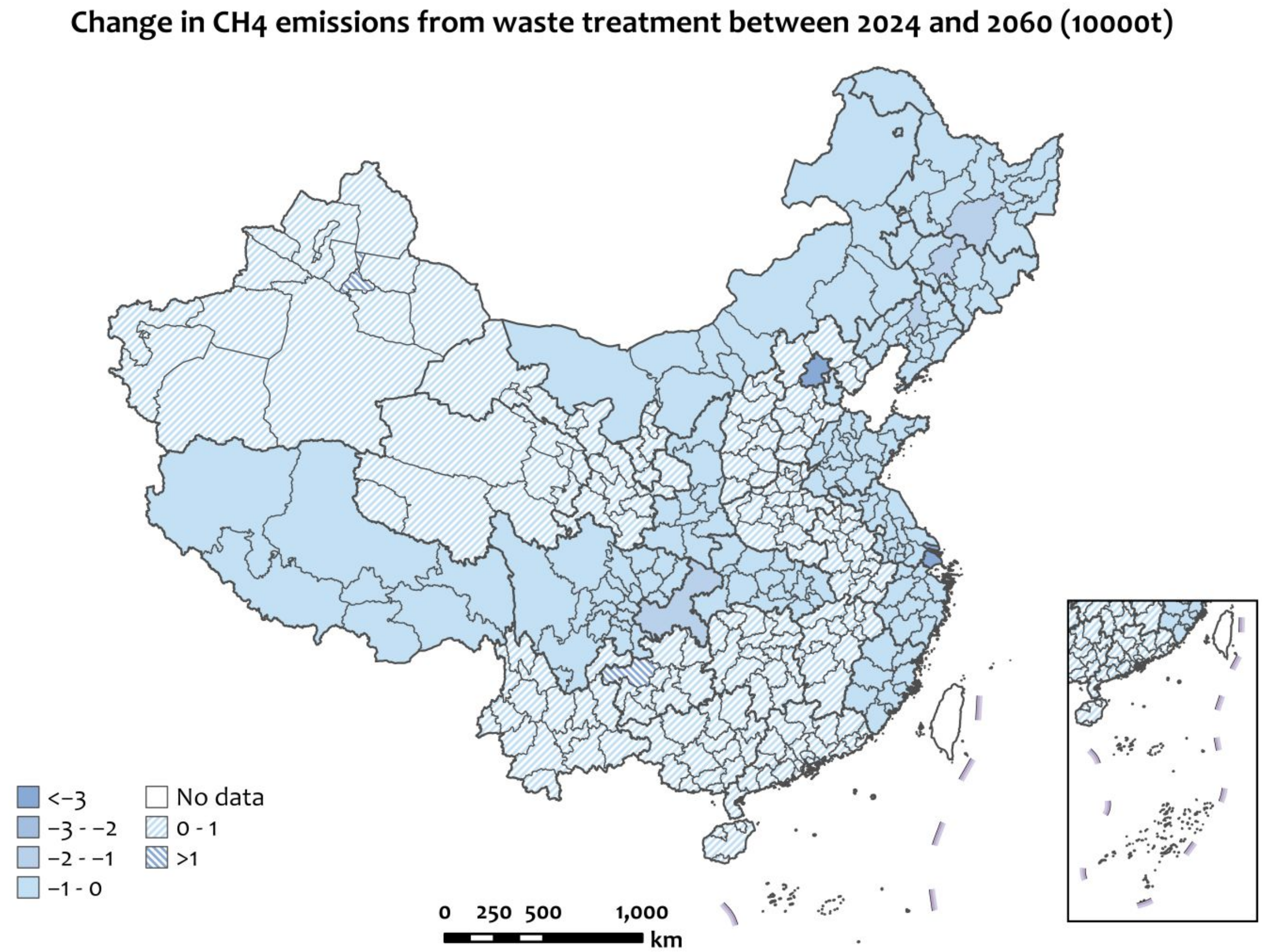

Fig. S5 | Projected changes in CH_4 emissions from waste treatment between 2024 and 2060 in Chinese prefecture-level cities.

Map showing city-level changes in methane emissions from waste treatment (landfills and wastewater treatment) between 2024 and 2060, expressed in 10,000 t (10^4 t). Blue shades denote emission reductions, from small declines (−1 to 0 ×10^4 t) to larger reductions (<−3 ×10^4 t). Hatched areas represent cities with projected slight or moderate increases (0–1 and >1 ×10^4 t), and white areas indicate no data. Overall, modest reductions dominate across most of China, with scattered pockets of increasing waste-sector emissions.

Table S11. The sources of activity levels and emission factors in the methane emission accounting of this study

	Activity level	Emission factor
**Energy sector**		
Coal production	CHRED Database, Wind Database, Global Energy Monitor, City Statistical Yearbook, Energy Balance Tables for Each Region	Provincial Greenhouse Gas Inventory Compilation Guidelines
Oil and gas production		
**Agricultural sector**		
Livestock and poultry farming	China Statistical Yearbook, China Rural Statistical Yearbook, China Animal Husbandry and Veterinary Yearbook, China Animal Husbandry Industry Statistical Data, MODIS NDVI data, Provincial Agricultural Statistical Yearbook, Wind Database	
Rice cultivation		
**Waste treatment sector**		
Sewage treatment	CHRED Database, China Environmental Statistical Yearbook, Wind Database	
Waste disposal	CHRED Database, China Urban Construction Statistical Yearbook, China Environmental Statistical Yearbook	

Table S12. Waste compositions in seven regions of China

	Provinces	Number of cities investigated	Composition of waste type with degradable contents (%)			
			Kitchen waste	Paper	Textile	Wood
Northwest China	Xinjiang, Gansu, Qinghai, Ningxia, Shaanxi	7	39.26	5.00	2.38	3.92
North China	Shanxi, Inner Mongolia, Hebei, Beijing, Tianjin	7	53.25	7.67	3.26	3.21
Northeast China	Heilongjiang, Jilin, Liaoning	9	59.9	7.30	1.54	2.30
Central China	Henan, Hubei, Hunan	6	35.57	4.58	1.07	1.73
East China	Shandong, Jiangsu, Anhui, Shanghai, Zhejiang, Fujian, Jiangxi	16	54.07	7.27	4.45	1.55
Southwest China	Chongqing, Sichuan, Guizhou, Yunnan, Tibet	6	45.54	7.14	3.14	3.64
South China	Guangdong, Guangxi, Hainan	4	44.05	5.80	2.00	3.17

Table S13. The wet-based ratios of degradable organic carbon in different components of MSW

Source	Kitchen waste	Paper	Textile	Wood
Results in this study	0.11	0.24	0.27	0.33
IPCC default value	0.15	0.40	0.24	0.43

Table 14. Key parameters in the FOD model

Type of landfill[a]	CH_4 oxidation factor			CH_4 correction factor	CH_4 recovery rate (%)
	Northwest	North China, Northeast China, Central China, East China	Southwest China, South China		
I	0	0	0	1	40
II	0.08	0.10	0.15	0.92	24
III	0.15	0.20	0.30	0.61	5

Note: [a] The landfills were categorized into three levels based on their sizes.

Table S15. Data sources for driving-force parameters used in LMDI

Parameter	Symbol	Definition	Unit	Years	Primary data source	Spatial level	Processing / notes
Population	P	Resident population/ Registered (hukou) population	persons	2018–2024	Provincial/prefecture-level Statistical Yearbooks; Statistical Communiqués on National Economic and Social Development (local statistical bureaus)	Prefecture-level city	City-code harmonization; missing-year values were carried forward using the previous year's data (last observation carried forward, LOCF), when necessary.
Economic activity	GDP	Gross Domestic Product (GDP)	CNY (constant price; base year = 2018)	2018–2024	Provincial/prefecture-level Statistical Yearbooks; Statistical Communiqués on National Economic and Social Development (local statistical bureaus)	Prefecture-level city	Deflated to constant prices using official deflators; units were harmonized across sources; missing-year values were carried forward using the previous year's data (LOCF), when necessary.
Sectoral structure	S	Sectoral structure derived from this study's sector-resolved inventory	%	2018–2024	Derived from this study's city-level, sector-resolved CH_4 emission inventory.	Prefecture-level city	Calculated as sectoral emission shares (sector emissions divided by total emissions) for each city-year; normalized to sum to 1; consistent sector definitions across years. Reported as three sectoral components in the Results and figures: energy activities, agricultural activities, and waste treatment.
Emission intensity	E	CH_4 emissions per unit	kg CH_4 / CNY	2018–2024	Derived from this study's city-level inventory + GDP	Prefecture-level city	Computed as M_i/A; consistent units ensured.

(derived)	activity (e.g., per GDP)	

Table S16. Projected energy consumption by 2060[9]

Type	2022	2030	2040	2050	2060
Coal (10^8t)	48.5	37.5-48.4	21-41.4	21-25.7	9.4-12
Petroleum (10^8t)	7.19	5.7-6.8	5.5-5.7	3.5-3.6	1.4-1.5
natural gas ($10^8 m^3$)	3663	3000-5200	2700-5500	2700-4500	1800-3000

Table S17. Emission reduction contribution of emission reduction technologies (%)[10]

Emission Reduction Technology	2030	2060
Compressor Supercharging Injection into Gas Transmission Pipeline Network	0.023	0.018
High-pressure Separation and Recovery Technology for Vent Gas	0.02	0.027
Testing and Maintenance of Pressure Safety Valves (PSV)	10	14.152
Small Skid-mounted Liquefied Natural Gas (LNG) Unit	0.015	0.017
CNG Recovery and Utilization Technology for Vent Gas	0.202	0.135
Hydrate-based Recovery and Utilization Technology for Vent Gas	0.081	0.04
Associated Gas Power Generation Technology	0.101	0.136
Targeted Inspection and Maintenance for Gas Processing Plants and Booster Stations	3.159	4.244
Natural Gas Power Generation Technology	0.018	0.022
Recovered Associated Gas for Boiler Combustion	0.125	0.1
Flare Gas Recovery (FGR) Technology	0.157	0.118
Heating Throttling and Compressor Supercharging Recovery Technology	1.097	1.447
Valve Leakage Repair during Pipeline Replacement	5.641	7.558

**Emission Reduction Technology**	**2030**	**2060**
Hydrocarbon Recovery Combined with Gas-fired Power Generation	1.558	1.165
Natural Gas Power Generation Technology	0.005	0.003
Installation of Flare Compressor Recovery System	0.006	0.003
Recovery and Utilization Technology for Fugitive Gas from Fracturing Flowback Fluid	0.04	0.048
CNG Natural Gas Storage and Transportation Technology	0.731	0.965
CNG Recovery Technology	8.001	9.572
Hydrocarbon Vapor Recovery from Storage Tanks	4.531	3.159
LNG Recovery of Vent Gas	0.004	0.004
CNG Recovery Technology for Flare Gas	0.362	0.478
Small Skid-mounted Light Hydrocarbon Recovery Unit	0.004	0.003
Purification Combined with Gas-fired Power Generation Technology	0.823	1.165
Low-pressure Flare Gas Compression Recovery Technology	0.065	0.081
Recovery Technology for Compressor Stations	3.084	3.689
Targeted Inspection and Maintenance for Gate Stations and Surface Facilities	2.418	3.239
Purification Combined with Skid-mounted CNG Recovery Technology	0.073	0.055
Booster Injection into Pipeline	0.559	0.449
Flare Gas Venting Recovery Technology	0.001	0.001
Purification Combined with Booster Re-injection Technology	1.829	2.588

**Emission Reduction Technology**	**2030**	**2060**
Purification Combined with Skid-mounted CNG Recovery Technology	0.291	0.384
Natural Gas Recovery from Fracturing Flow-back	4.389	5.787
Mobile CNG Technology	0.019	0.021
Purification-Hydrocarbon Recovery-Skid-mounted CNG Recovery Integrated Technology	0.183	0.241
Gas Storage Well Recovery Technology	0.64	0.906
Mobile Compressor Recovery Technology	9.251	11.067
Condensation-based Oil and Gas Recovery Technology	1.726	0.518
Suspension Pumping Low-pressure Casing Gas Recovery Technology	0.693	1.256
Hydrocarbon Recovery Combined with CNG Compression Technology	0.438	0.314
Casing Gas Substitution for Electric Heaters	0.197	0.158
Associated Gas Recovery Technology for Scattered Pilot Production Wells	0.004	0.003
Fugitive Gas Recovery from Open Liquid Surface	0.02	0.015
Integrated Flash Gas Recovery Unit	0.718	0.965
LDAR (Leak Detection and Repair) for Urban Gas Distribution	4.955	6.638
LDAR for Oil and Gas Processing Stations	0.257	0.151
LDAR for Oil and Gas Processing Stations	4.119	3.216
LDAR for Oil and Gas Wellheads	5.589	4.486

Table S18. Predictions for rice cultivation by 2060[11–13]

Type	2022	2030	2035	2060
Single-season rice(10^4t)	12887.2	13200-13500	12900-13200	13000-13800
Double-season rice (10^4t)	7962.3	7800-8000	7600-7800	8000-8500

Table S19. Predictions for livestock farming by 2060[13]

Commodity	Total Production (Mt)				
	2018	2030	2040	2050	2060
Pork	54	60	61.6	61.4	58.7
Beef	6.4	7.9	8.7	9.4	9.4
Mutton	4.8	5.8	6.8	6.8	6.8
Poultry Meat	23.6	25.6	27.7	30	32.5
Poultry Eggs	31.3	36.5	40.2	44.2	44.2
Milk	30.4	43.9	45	45.6	45.6

Table S20. Predictions for livestock farming by 2060[13]

Commodity	Total Production (Mt)				
	2018	2030	2040	2050	2060
Pork	54	60	61.6	61.4	58.7
Beef	6.4	7.9	8.7	9.4	9.4
Mutton	4.8	5.8	6.8	6.8	6.8
Poultry Meat	23.6	25.6	27.7	30	32.5
Poultry Eggs	31.3	36.5	40.2	44.2	44.2
Milk	30.4	43.9	45	45.6	45.6

Table S21. Emission reduction contribution of emission reduction technologies (%)[4]

	Enteric fermentation		Manure management	Rice cultivation			Others			
Province	M1	M2	M3	M4	M5	M6	M7	M8	M9	M10
Beijing	22.00	23.00	62.60	80.00	18.80	30.00	29.80	3.80	62.70	5
Tianjin	20.10	23.00	62.60	80.00	18.80	30.00	29.80	3.50	54.10	5
Hebei	22.90	23.00	60.30	78.60	18.80	30.00	29.80	4.60	49.50	5
Shanxi	27.80	23.00	58.10	75.50	18.80	30.00	29.80	3.60	65.10	5
Inner Mongolia	25.50	23.00	52.80	68.90	18.80	30.00	29.80	5.30	51.00	5
Liaoning	21.80	23.00	63.90	78.10	18.80	30.00	29.80	5.00	74.30	5
Jilin	19.60	23.00	62.60	75.70	18.80	30.00	29.80	4.40	70.10	5
Heilongjiang	19.70	23.00	62.10	75.00	18.80	30.00	29.80	4.80	62.80	5
Shanghai	21.00	23.00	69.80	86.20	18.80	30.00	29.80	4.10	68.50	5
Jiangsu	26.30	23.00	70.60	85.70	18.80	30.00	29.80	4.30	57.90	5
Zhejiang	26.70	23.00	70.40	87.00	18.80	30.00	29.80	4.80	61.40	5
Anhui	23.40	23.00	69.50	85.50	18.80	30.00	29.80	4.50	68.40	5
Fujian	19.90	23.00	71.00	84.30	18.80	30.00	29.80	4.30	63.00	5
Jiangxi	18.20	23.00	68.20	86.60	18.80	30.00	29.80	5.20	60.50	5
Shandong	23.30	23.00	60.50	79.80	18.80	30.00	29.80	3.90	73.70	5
Henan	21.80	23.00	59.80	80.30	18.80	30.00	29.80	4.00	55.60	5
Hubei	20.60	23.00	68.90	85.60	18.80	30.00	29.80	4.40	56.10	5
Hunan	19.80	23.00	69.40	86.60	18.80	30.00	29.80	5.00	62.50	5
Guangdong	17.10	23.00	68.60	87.90	18.80	30.00	29.80	4.20	70.20	5
Guangxi	18.10	23.00	66.00	86.50	18.80	30.00	29.80	4.60	60.00	5
Hainan	17.80	23.00	65.60	87.70	18.80	30.00	29.80	4.00	64.80	5
Chongqing	20.40	23.00	69.10	86.70	18.80	30.00	29.80	5.60	57.20	5
Sichuan	19.70	23.00	66.30	83.70	18.80	30.00	29.80	5.10	79.60	5
Guizhou	18.90	23.00	63.90	82.10	18.80	30.00	29.80	4.10	72.40	5

Yunnan	20.70	23.00	66.10	84.50	18.80	30.00	29.80	4.60	58.50	5
Tibet	20.20	23.00	47.80	59.70	18.80	30.00	29.80	4.90	64.60	5
Shaanxi	24.50	23.00	59.40	78.10	18.80	30.00	29.80	3.70	68.80	5
Gansu	23.90	23.00	49.50	68.40	18.80	30.00	29.80	4.10	52.00	5
Qinghai	21.30	23.00	49.00	58.30	18.80	30.00	29.80	3.90	57.60	5
Ningxia	24.60	23.00	48.30	67.50	18.80	30.00	29.80	3.20	54.60	5
Xinjiang	25.70	23.00	50.00	69.60	18.80	30.00	29.80	3.80	45.30	5

Note: M1 (Increasing fat content in livestock diets), M2 (Ensiling, ammonization and palettization of straw), M3 (Acidification treatment of manure), M4 (Composting + Anaerobic Digestion), M5 (Breeding new rice varieties), M6(Intermittent Irrigation), M7 (Improving rice fertilization management), M8 (Reducing food waste), M9 (Adjusting dietary structure), M10 (Incentive-type emission reduction policies.

Table S22. Predictions for livestock farming by 2060 (Mt)[14]

**Unit: Mt**	**2020**	**2025**	**2030**	**2050**	**2060**
Industrial Wastewater COD Generation	33	36	42	53	58
Domestic Wastewater BOD Generation	21	23	21	19	12

Note: The data for the year 2060 is estimated based on the average growth rate from 2020 to 2050.

Table S23. The forecasted landfilled MSW generation by 2060 based on SSP (Kt)[15]

	**SSP1**	**SSP2**	**SSP3**	**SSP4**	**SSP5**
2025	5.3855	5.3987	5.3656	5.3644	5.4063
2030	5.5662	5.5788	5.5041	5.5141	5.6116
2035	5.7093	5.7094	5.5912	5.6091	5.7769
2040	5.8148	5.7976	5.6436	5.6597	5.9020
2050	5.9264	5.8767	5.6599	5.6814	6.0282
2060	6.0123	5.9876	5.8345	5.8534	6.1324

Note: The data for the year 2060 is estimated based on the average growth rate from 2025 to 2050.

Table S24. Emission reduction contribution of emission reduction technologies (%)[15]

	LFG utilization	Organic waste treatment	Landfill renovation	Half waste
2025	-5	-6	-4	-5
2030	-26	-29	-23	-27
2035	-34	-39	-30	-36
2040	-40	-47	-34	-43
2050	-52	-64	-43	-57
2060	-53	-67	-44	-59

Note: Landfill renovation (Functional soil covers, Renewable landfill). LFG utilization (LFG collection and combustion, LFG collection for power generation, LFG purification and utilization). Organic waste treatment (Composting, Anaerobic digestion).

Table S25. Projected population of each province by 2060 (10,000 persons)[16]

Province	2025	2030	2035	2040	2050	2060
Beijing	2207	2180	2129	2065	1920	1704
Tianjin	1412	1408	1386	1354	1280	1171
Hebei	7582	7592	7564	7530	7391	7082
Shanxi	3452	3420	3394	3493	3349	3121
Inner Mongolia	2432	2424	2392	2344	2204	2004
Liaoning	4245	4176	4059	3902	3499	3039
Jilin	2379	2325	2247	2148	1899	1605
Heilongjiang	3143	3072	2967	2836	2508	2122
Shanghai	2480	2436	2368	2287	2087	1814
Jiangsu	8504	8406	8252	8075	7615	6911

Province	2025	2030	2035	2040	2050	2060
Zhejiang	6524	6489	6396	6264	5879	5258
Anhui	6231	6269	6268	6258	6174	5891
Fujian	4239	4253	4236	4200	4052	3737
Jiangxi	4642	4716	4762	4783	4731	4553
Shandong	10439	10669	10626	9832	9302	8530
Henan	10268	10470	10573	10618	10607	10426
Hubei	5831	5812	5747	5671	5470	5130
Hunan	6757	6795	6798	6783	6654	6354
Guangdong	13037	13411	13633	13193	12995	12307
Guangxi	5230	5387	5516	5635	5828	5894
Hainan	1066	1104	1136	1166	1217	1245
Chongqing	3238	3225	3181	3118	2961	2756
Sichuan	8390	8308	8153	7944	7418	6734
Guizhou	4065	4219	4325	4421	4646	4823
Yunnan	4859	4939	4977	4991	4936	4735
Xizang	377	385	388	388	373	342
Shaanxi	4002	3977	3910	3821	3600	3289
Gansu	2560	2576	2563	2538	2459	2314

Province	2025	2030	2035	2040	2050	2060
Qinghai	609	618	620	618	597	559
Ningxia	756	781	798	811	820	811
Xinjiang	2759	2897	3019	3139	3347	3543

Table S26. Projected income distribution of each province by 2060 (thousand yuan)[17]

Province	2025	2030	2035	2040	2050	2060
Beijing	11.27551	11.49265	11.66928	11.77045	11.80238	11.83276
Tianjin	10.69879	11.06229	11.40611	11.66267	11.93375	12.06425
Hebei	9.955477	10.22632	10.47627	10.69624	11.02043	11.25156
Shanxi	9.816821	10.02886	10.25274	10.44464	10.73197	10.90827
Inner Mongolia	10.15321	10.41437	10.64142	10.83133	11.11953	11.35559
Liaoning	10.30157	10.56358	10.81488	11.05127	11.4372	11.74154
Jilin	9.987984	10.33002	10.60491	10.84932	11.21222	11.44706
Heilongjiang	9.903543	10.16613	10.40364	10.57746	10.89996	11.12692
Shanghai	11.08421	11.33329	11.55245	11.72067	11.88313	12.09015
Jiangsu	10.62328	10.87198	11.07159	11.24817	11.52069	11.75886
Zhejiang	10.77601	11.00828	11.21186	11.38325	11.64005	11.92059
Anhui	9.968616	10.12727	10.23974	10.31939	10.46443	10.62161
Fujian	10.44879	10.72628	10.94095	11.09113	11.29473	11.48665
Jiangxi	9.976391	10.17394	10.32329	10.43145	10.61475	10.79937
Shandong	10.35716	10.64811	10.89204	11.09782	11.42097	11.67694
Henan	9.951817	10.21364	10.44377	10.63992	10.97318	11.23418
Hubei	10.03188	10.23112	10.36942	10.48753	10.67097	10.80897
Hunan	10.03551	10.23241	10.372	10.48557	10.69467	10.92724
Guangdong	10.47294	10.7033	10.91287	11.08001	11.30325	11.48091

Guangxi	9.734788	9.839353	9.923697	9.985304	10.05438	10.19334
Hainan	9.95847	10.13938	10.3029	10.42957	10.58424	10.74507
Chongqing	10.23579	10.41584	10.54766	10.62966	10.86077	11.02959
Sichuan	10.00465	10.261	10.44597	10.58664	10.91625	11.16302
Guizhou	9.602433	9.726976	9.816401	9.876344	9.951188	10.04553
Yunnan	9.577339	9.689376	9.755445	9.788845	9.84344	9.972032
Xizang	9.384948	9.531317	9.672732	9.81034	10.06569	10.27513
Shaanxi	9.959065	10.17919	10.38193	10.54108	10.79416	10.93274
Gansu	9.538854	9.812273	10.03928	10.24773	10.62519	10.89387
Qinghai	9.756025	10.00525	10.23727	10.42275	10.75114	11.04335
Ningxia	9.896084	10.09084	10.27756	10.41572	10.65797	10.9023
Xinjiang	9.829784	10.01088	10.15857	10.26061	10.40978	10.5631

Table S27. Assumed methane mitigation pathways for the energy, agriculture, and waste sectors across different periods

Period	Energy sector	Agricultural sector	Waste disposal sector
2025—2030	Low-concentration gas utilization, China's certificated emission reduction (CCER) methodology, associated gas recovery, LDAR inspection	Intermittent irrigation, low-protein diet, biogas project	Landfill gas recovery, incineration modification, sludge anaerobic digestion
2031—2035	Integrated monitoring of the sky and the earth, closing of mine pumping operations, green well completion	Biochar soil improvement, low-emission breeding, centralized biogas	Waste kitchen residue anaerobic digestion, collaborative treatment, and near-zero emission pilot project
2036—2060	Ultra-low concentration oxidation, satellite remote sensing verification, ecological restoration control	Genetic editing breeding, cell-cultured meat substitution, and consumption-side transformation	Comprehensive resource utilization, collaborative anaerobic digestion, negative emission technology

References

(1) Pagani, F.; Crippa, M.; Guizzardi, D.; Pisoni, E. GHG Emissions at Sub-National Level, 2023. https://doi.org/10.2905/D67EEDA8-C03E-4421-95D0-0ADC460B9658.

(2) Cui, D.; Yang, S.; Song, X.; Huang, X.; Duan, C.; Ji, M.; Li, Z.; Yu, S.; Deng, Z.; Ke, P.; Dou, X.; Sun, T.; Liu, Z. Monthly Methane Emissions in Chinese Mainland Provinces from 2013–2022. *Sci Data* **2025**, *12* (1), 948. https://doi.org/10.1038/s41597-025-05107-4.

(3) Liu, W.; Li, N.; Liu, L.; Gao, P.; Di, X.; Li, G. K. Methane Emissions from Landfills in China: Historical Trends, Future Trajectories, and Mitigation Strategies. *Resources, Conservation and Recycling* **2026**, *225*, 108625. https://doi.org/10.1016/j.resconrec.2025.108625.

(4) Duan, Y.; Gao, Y.; Zhao, J.; Xue, Y.; Zhang, W.; Wu, W.; Jiang, H.; Cao, D. Agricultural Methane Emissions in China: Inventories, Driving Forces and Mitigation Strategies. *Environ. Sci. Technol.* **2023**, *57* (36), 13292–13303. https://doi.org/10.1021/acs.est.3c04209.

(5) Liu, W.; Li, N.; Gao, P.; Di, X.; Liu, L.; Li, G. K. Tracking China's Methane Emissions from Wastewater: Inventories, Driving Forces, and Mitigation Potential. *Resources, Conservation and Recycling* **2024**, *210*, 107831. https://doi.org/10.1016/j.resconrec.2024.107831.

(6) Khanna, N.; Lin, J.; Liu, X.; Wang, W. An Assessment of China's Methane Mitigation Potential and Costs and Uncertainties through 2060. *Nat Commun* **2024**, *15* (1). https://doi.org/10.1038/s41467-024-54038-y.

(7) Lin, J.; Khanna, N.; Liu, X.; Teng, F.; Wang, X. China's Non-CO2 Greenhouse Gas Emissions: Future Trajectories and Mitigation Options and Potential. *Sci Rep* **2019**, *9* (1), 16095. https://doi.org/10.1038/s41598-019-52653-0.

(8) Wang, Y.; Zhu, Z.; Dong, H.; Zhang, X.; Wang, S.; Gu, B. Mitigation Potential of Methane Emissions in China's Livestock Sector Can Reach One-Third by 2030 at Low Cost. *Nat Food* **2024**, *5* (7), 603–614. https://doi.org/10.1038/s43016-024-01010-0.

(9) GE Shirong; FAN Jingli; LIU Shuqin; SONG Mei; XIAN Yujiao; WANG Bing; TENG Teng. Low Carbon Modern Coal-Based Energy Technology System and Development Strategy. *Journal of China Coal Society* **2024**, *49* (1), 203–223. https://doi.org/10.13225/j.cnki.jccs.2023.1773.

(10) Li, X.; Cheng, X.; Xue, M.; Wang, Y.; Jia, G.; Yan, Y.; Sun, J.; Jia, Y. Estimation and Reduction Analysis of Methane Emissions in China's Oil and Gas Industry. *Natural Gas Industry* **2024**, *44* (4), 160–170.

(11) Ministry of Agriculture and Rural Affairs of the People's Republic of China. *China Agricultural Outlook Report (2026-2035)*; Ministry of Agriculture and Rural Affairs of the People's Republic of China, 2026. https://scs.moa.gov.cn/gzdt/202604/t20260429_6483787.htm.

(12) LI, M.; GAO, X.; PAN, J.; XIONG, W.; GUO, L.; LIN, E.; LI, K. Possible Trends of Rice Yield in China under Global Warming by 1.5 ℃ and 2.0 ℃. *JOURNAL OF NATURAL RESOURCES* **2021**, *36* (3), 567–581. https://doi.org/10.31497/zrzyxb.20210303.

(13) Chen, M.; Cui, Y.; Jiang, S.; Forsell, N. Toward Carbon Neutrality before 2060: Trajectory and Technical Mitigation Potential of Non-CO2 Greenhouse Gas Emissions from Chinese Agriculture. *Journal of Cleaner Production* **2022**, *368*, 133186. https://doi.org/10.1016/j.jclepro.2022.133186.

(14) Khanna, N.; Lin, J.; Liu, X.; Wang, W. An Assessment of China's Methane Mitigation

Potential and Costs and Uncertainties through 2060. *Nat Commun* **2024**, *15* (1), 9694. https://doi.org/10.1038/s41467-024-54038-y.
(15) Liu, W.; Li, N.; Liu, L.; Gao, P.; Di, X.; Li, G. K. Methane Emissions from Landfills in China: Historical Trends, Future Trajectories, and Mitigation Strategies. *Resources, Conservation and Recycling* **2026**, *225*, 108625. https://doi.org/10.1016/j.resconrec.2025.108625.
(16) Chen, Y.; Guo, F.; Wang, J.; Cai, W.; Wang, C.; Wang, K. Provincial and Gridded Population Projection for China under Shared Socioeconomic Pathways from 2010 to 2100. *Sci Data* **2020**, *7* (1), 83. https://doi.org/10.1038/s41597-020-0421-y.
(17) Lei, M.; Pelz, S.; Pachauri, S.; Cai, W. A Dataset of Income Distribution on Provincial, Urban, and Rural Levels for China from 2020 to 2100. *Sci Data* **2024**, *11* (1), 1436. https://doi.org/10.1038/s41597-024-04304-x.
